# *RBCS1 *expression in coffee: *Coffea *orthologs, *Coffea arabica *homeologs, and expression variability between genotypes and under drought stress

**DOI:** 10.1186/1471-2229-11-85

**Published:** 2011-05-16

**Authors:** Pierre Marraccini, Luciana P Freire, Gabriel SC Alves, Natalia G Vieira, Felipe Vinecky, Sonia Elbelt, Humberto JO Ramos, Christophe Montagnon, Luiz GE Vieira, Thierry Leroy, David Pot, Vânia A Silva, Gustavo C Rodrigues, Alan C Andrade

**Affiliations:** 1 Embrapa Recursos Genéticos e Biotecnologia (LGM-NTBio), Parque Estação Biológica, CP 02372, 70770-917 Brasilia, Distrito Federal, Brazil; 2 CIRAD UMR AGAP, 34398 Montpellier Cedex 5, France; 3 Instituto Agronômico do Paraná (IAPAR/LBI-AMG), Rodovia Celso Garcia Cid, Km 375, CP 481, 86001-970 Londrina, Paraná, Brazil; 4 Universidade Federal de Viçosa (UFV), PH Rolfs S/A, 36570-000 Viçosa, Minas Gerais, Brazil; 5 CIRAD UMR RPB, 34398 Montpellier Cedex 5, France; 6 EPAMIG/URESM, Rodovia Lavras/IJACI, Km 02, CP 176, 37200-000 Lavras, Minas Gerais, Brazil; 7 Embrapa Cerrados, BR 020 Km18, CP 08223, 73310-970 Planaltina, Distrito Federal, Brazil

## Abstract

**Background:**

In higher plants, the inhibition of photosynthetic capacity under drought is attributable to stomatal and non-stomatal (i.e., photochemical and biochemical) effects. In particular, a disruption of photosynthetic metabolism and Rubisco regulation can be observed. Several studies reported reduced expression of the *RBCS *genes, which encode the Rubisco small subunit, under water stress.

**Results:**

Expression of the *RBCS1 *gene was analysed in the allopolyploid context of *C. arabica*, which originates from a natural cross between the *C. canephora *and *C. eugenioides *species. Our study revealed the existence of two homeologous *RBCS1 *genes in *C. arabica*: one carried by the *C. canephora *sub-genome (called *CaCc*) and the other carried by the *C. eugenioides *sub-genome (called *CaCe*). Using specific primer pairs for each homeolog, expression studies revealed that *CaCe *was expressed in *C. eugenioides *and *C. arabica *but was undetectable in *C. canephora*. On the other hand, *CaCc *was expressed in *C. canephora *but almost completely silenced in non-introgressed ("pure") genotypes of *C. arabica*. However, enhanced *CaCc *expression was observed in most *C. arabica *cultivars with introgressed *C. canephora *genome. In addition, total *RBCS1 *expression was higher for *C. arabica *cultivars that had recently introgressed *C. canephora *genome than for "pure" cultivars. For both species, water stress led to an important decrease in the abundance of *RBCS1 *transcripts. This was observed for plants grown in either greenhouse or field conditions under severe or moderate drought. However, this reduction of *RBCS1 *gene expression was not accompanied by a decrease in the corresponding protein in the leaves of *C. canephora *subjected to water withdrawal. In that case, the amount of RBCS1 was even higher under drought than under unstressed (irrigated) conditions, which suggests great stability of RBCS1 under adverse water conditions. On the other hand, for *C. arabica*, high nocturnal expression of *RBCS1 *could also explain the accumulation of the RBCS1 protein under water stress. Altogether, the results presented here suggest that the content of RBCS was not responsible for the loss of photosynthetic capacity that is commonly observed in water-stressed coffee plants.

**Conclusion:**

We showed that the *CaCe *homeolog was expressed in *C. eugenioides *and non-introgressed ("pure") genotypes of *C. arabica *but that it was undetectable in *C. canephora*. On the other hand, the *CaCc *homeolog was expressed in *C. canephora *but highly repressed in *C. arabica*. Expression of the *CaCc *homeolog was enhanced in *C. arabica *cultivars that experienced recent introgression with *C. canephora*. For both *C. canephora *and *C. arabica *species, total *RBCS1 *gene expression was highly reduced with WS. Unexpectedly, the accumulation of RBCS1 protein was observed in the leaves of *C. canephora *under WS, possibly coming from nocturnal *RBCS1 *expression. These results suggest that the increase in the amount of RBCS1 protein could contribute to the antioxidative function of photorespiration in water-stressed coffee plants.

## Background

With a world production of 134 million bags of beans in 2010 http://www.ico.org, coffee is the most important agricultural commodity worldwide and a source of income for many developing tropical countries [1]. In the genus *Coffea*, two species are responsible for almost all coffee bean production: *Coffea canephora *and *Coffea arabica*, which contribute approximately 30 and 70% of worldwide production, respectively [2]. *C. canephora *is a diploid (2n = 2x = 22) and allogamous *Coffea *species. On the other hand, *C. arabica *is an amphidiploid (allotetraploid, 2n = 4x = 44), which comes from a natural hybridisation estimated to have taken place more than 100,000 years ago between the ancestors of present-day *C. canephora *and *C. eugenioides *[3]. In this context, the transcriptome of *C. arabica *is a mixture of homeologous genes expressed from these two sub-genomes [4]. Aside from the pure "Arabica" varieties, *C. arabica *cultivars recently introgressed with *C. canephora *genome have been selected in order to take advantage of available *C. canephora*'s disease-resistant genes. Natural and recent interspecific (*C. arabica *x *C. canephora*) Timor Hybrids as well as controlled interspecific crosses provided the progenitors for these introgressed *C. arabica *varieties [5].

Coffee production is subjected to regular oscillations explained mainly by the natural biennial cycle but also by the adverse effects of climatic conditions. Among them, drought and high temperature are key factors affecting coffee plant development and production [6,7]. If severe drought periods can lead to plant death, moderate drought periods are also very damaging to coffee growers by affecting flowering, bean development and, consequently, coffee production. In addition, large variations in rainfall and temperature also increase bean defects, modify bean biochemical composition and the final quality of the beverage [8-11]. As a result of global climate change, periods of drought may become more pronounced, and the sustainability of total production, productivity and coffee quality may become more difficult to maintain [12].

The primary effects of water stress (WS) on physiological and biochemical processes in plants have been extensively discussed [13-16]. They are attributable to various processes, including diffusional (stomatal and mesophyllian resistances to the diffusion of CO_2_), photochemical (regulation of light harvest and electron transport) and/or biochemical processes (*e.g.*, regulation of ribulose-1,5-bisphosphate carboxylase/oxygenase content or activity and regulation of the Calvin cycle through exports of assimilates). Stomatal closure is one of the earliest responses to short-term soil drying, therefore limiting water loss and net carbon assimilation (A) by photosynthesis. The decrease of photosynthesis under WS can come from CO_2 _limitation mediated by stomatal closure or by a direct effect on the photosynthetic capacity of chloroplasts. Independently of the nature of this reduction, the intensity of the intercepted irradiance can greatly exceed the irradiance necessary to saturate photosynthesis. As CO_2 _assimilation precedes inactivation of electron transfer reactions, an excess of reducing power is frequently generated in water-stressed plants [17]. Thus, this excess can be used to reduce the molecular oxygen leading to the formation of reactive oxygen species (ROS) and causing photooxidative damage [18]. Under prolonged drought stress, reduced growth, reduced leaf area and altered assimilate partitioning among tree organs seems to be responsible for decreased crop yield [19]. In C_3 _plants, the key photosynthetic enzyme is the Rubisco (ribulose-1,5-bisphosphate carboxylase/oxygenase, EC 4.1.1.39), which is responsible for CO_2 _fixation and photorespiration [20]. This enzyme is localised in the chloroplast stroma and accounts for approximately 30-60% of the total soluble protein in plants. Rubisco also constitutes a large pool of stored leaf nitrogen that can be quickly remobilised under stress and senescence [21,22]. In higher plants, the Rubisco holoenzyme is composed of large (RBCL) and small (RBCS) subunits encoded respectively by the unique chloroplastic *RBCL *gene and the small *RBCS *multigene family located in the nucleus [23]. In fact, potential Rubisco activity is determined by the amount of Rubisco protein, which in turn is determined by the relative rate of biosynthesis and degradation. These processes are regulated by gene expression, mRNA stability, polypeptide synthesis, post-translational modification, assembly of subunits into an active holoenzyme, and various factors that impact upon protein degradation [24-26].

Numerous studies have shown that *RBCS *transcripts accumulate differentially in response to light intensity or tissue development [for a review, see [27]]. This raises the possibility that RBCS subunits may regulate the structure or function of Rubisco [28]. At the molecular level, drought stress suppresses the expression of many photosynthetic genes including the *RBCS *genes [29-33]. In contrast, transcripts encoding enzymes of the pentose phosphate and glycolytic pathway (*e.g.*, glucose-6-phosphate dehydrogenase and pyruvate kinase) were induced during drought, suggesting that these pathways are used for the production of reducing power in the absence of photosynthesis during stress [34]. Even if Rubisco inactivation contributes to the non-stomatal limitation of photosynthesis under drought stress [35,36], data demonstrated a Rubisco reduction in stressed plants [37-39]. This is in agreement with the observation that part of the biochemical limitation of the photosynthetic rate (*A*) during drought comes from Rubisco regeneration rather than from a decrease in Rubisco activity [40]. In that sense, the WS-induced decrease in Rubisco content may characterise a general stimulation of senescence and/or the specific degradation of this protein by oxidative processes [41]. However, other work has reported that the amount of Rubisco protein is poorly affected by moderate and even prolonged severe drought [42]. The mechanism by which Rubisco may be down-regulated due to tight binding inhibitors could be pivotal for the tolerance and recovery from stress [38]. Rubisco binding proteins that are able to stabilise Rubisco could also be related to drought tolerance [41,43], but their roles in the structure, function and regulation of RBCS subunits are poorly understood [28,44].

During the last decade, coffee breeding programs identified clones of *C. canephora *var. Conilon that presented differential responses to WS [45]. Physiological characteristics of these clones revealed differences in root depth, stomatal control of water use and long-term water use efficiencies (WUE), which were estimated through carbon isotope discrimination [for a review, see [7]]. Even if some coffee cultivars perform osmotic adjustment under water deficit stress [46], little is known about the mechanisms of drought stress tolerance in coffee trees [47]. When studying container-grown *C. arabica *L. plants for 120 days under three soil moisture regimes, Meinzer *et al. *[48] observed that the total leaf area of plants irrigated twice a week was one-half that of plants irrigated twice a day although their assimilation rates on a unit-leaf-area basis were nearly equal throughout the experiment. This suggests that the maintenance of nearly constant photosynthetic characteristics on a unit-leaf-area basis through the maintenance of a smaller total leaf area may constitute a major mode of adjustment to reduced soil moisture availability in coffee. Similar results were also reported for field-grown *C. canephora *[46].

The periodicity of coffee vegetative growth is also heavily dependent on several environmental factors, such as temperature, photoperiod, irradiance and water supply. Seasonal changes in vegetative growth and photosynthesis were previously reported for field-grown plants of *C. arabica *L. cv. Catuaí Vermelho [49]. In that case, the reduced growth period during the winter season was characterised by a decline in air temperature leading to a decrease in the net carbon assimilation rate (*A*) and leaf starch accumulation. This decrease in photosynthesis during the winter season is not likely to be due to stomatal limitation because *g*_s _(stomatal conductance) remains relatively high at the same time. Kanechi *et al. *[50] showed that low rates of photosynthesis were accompanied by a decreased content of Rubisco in coffee leaves exposed to prolonged WS. In another study, Kanechi *et al. *[51] also demonstrated that leaf photosynthesis in coffee plants exposed to rapid dehydration decreased as a consequence of non-stomatal limitation that was associated with the inhibition of Rubisco activity.

Regarding the importance of photosynthesis in controlling plant development and the lack of information concerning expression of genes coding for Rubisco subunits in coffee, here, we decided to first focus on the expression of *RBCS1 *genes encoding the small subunit of Rubisco. Using the recent advances in coffee genomics [52-57] and the *CaRBCS1 *cDNA available from *C. arabica *[58], our study aims to (i) identify the different coffee *RBCS1 *gene homeologs corresponding to the *C. canephora *and *C. eugenioides *ancestor sub-genomes of the amphidiploid *C. arabica *species, (ii) evaluate the expression of these alleles in different coffee genotypes and species with an emphasis on *C. arabica *cultivars with and without recent introgression from *C. canephora *and (iii) study the effects of different (moderate and severe) WS on *RBCS1 *expression in juvenile and adult *C. canephora *and *C. arabica *plants. Finally, *RBCS1 *expression was also studied at different times of the day and discussed in relation to the RBCS1 protein profiles observed under WS.

## Results

### Identification of coffee cDNA sequences coding for RBCS1 (ribulose-1,5-bisphosphate carboxylase/oxygenase small subunit)

**Figure 1:**
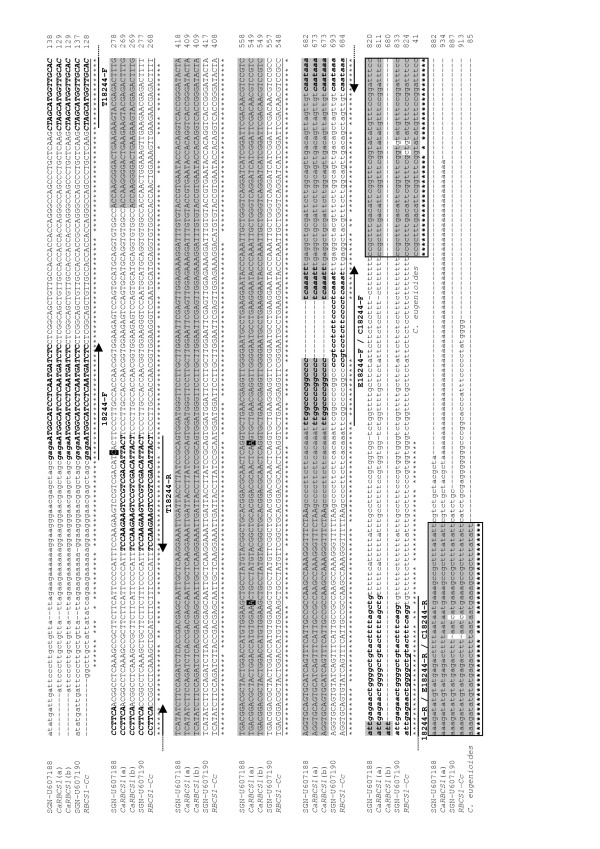
**Alignment of coffee *RBCS1 *nucleic sequences**. Sequences of the *CaRBCS1 *cDNA [58] from *C. arabica *cv. Caturra (a) [Genbank:AJ419826] and of the corresponding gene (b) [GenBank:AJ419827] without introns, were aligned with the unigenes SGN-U607188, SGN-U607190 and *RBCS1-Cc *(identical to SGN-U617577 formed by the alignment of 145 reads found in leaf cDNA libraries from *C. canephora*) from the SOL genomic database [56] and with the unique *RBCS1 *homologous read of *C. eugenioides *[4]. The SGN-U607188 and SGN-U607190 unigenes were formed by the alignment of reads found in cDNA libraries from fruits and the leaves of *C. arabica *plants. The coding sequences of the partial *RBCS1 *genes from genotypes of *C. arabica *[Genbank:DQ300266 to DQ300277; L.S. Ramirez, unpublished results] that matched with *CaRBCS1 *sequences are underlined in grey, while base differences are boxed in black. The *CcRBCS1 *cDNA sequence [GenBank:FR728242, this work] corresponded to the underlined sequence of the SGN-U617577 unigene. For all the sequences, the coding sequence is in uppercase, and the 5' and 3' UTR regions are in lower case. Horizontal arrows as well as nucleotides in bold and italics indicate the primers (Table 1) used for qPCR reactions. The stars below the alignments indicate identical bases, and the nucleotides are numbered for each lane.

The use of the *CaRBCS1 *[GenBank:AJ419826] cDNA from *C. arabica *as a query sequence identified several similar sequences in the coffee databases, and they were aligned for comparison (Figure 1). The *C. arabica *unigene SGN-U607188 preferentially aligned with the *CaRBCS1 *cDNA and gene sequences already reported for this species, and it matched perfectly with the coding sequences of partial *RBCS1 *genes cloned from different genotypes of *C. arabica *[GenBank:DQ300266 to DQ300277; L.S. Ramirez, unpublished results]. On the other hand, the *C. arabica *unigene (SGN-U607190) was more identical to the *C. canephora *SGN-U617577 unigene than other *C. arabica *SGN-U607188 unigene. A single and short *RBCS1 *EST of *C. eugenioides *[4] was also aligned with these sequences. Notably, it was strictly identical with the *CaRBCS1 *and SGN-U607188 sequences from *C. arabica *but diverged by few bases with the unigenes SGN-U607190 and SGN-U617577 of *C. canephora*.

Within the RBCS1 protein-coding sequence, five bases differed between SGN-U607188 and SGN-U607190, but only three diverged between the sequences of *C. arabica*. The main difference between all of these sequences was found in their 3' untranslated (UTR) region by the presence of a 12-bp sequence (GTCCTCTTCCCC) localised 31 bp after the stop codon of the unigenes SGN-U607190 and SGN-U617577 of *C. canephora*, which was not observed in the *CaRBCS1 *gene and cDNA sequences. In addition, the *C. arabica *unigene SGN-U607190 was more related to the *C. canephora *unigene SGN-U617577 than to the previously-cloned *CaRBCS1 *cDNA.

*RBCS1 *cDNAs were sequenced from the Rubi (Mundo Novo x Catuaí) cultivar of *C. arabica *that did not recently introgress with *C. canephora *genomic DNA and clone 14 of *C. canephora *var. Conilon using primer pair 18244, which was designed to conserved *RBCS1 *cDNA regions of the two species. For the Rubi cultivar, the cDNA was strictly identical to the RBCS1 coding region of the *CaRBCS1 *gene [GenBank:AJ419827] and without detection of any single nucleotide polymorphisms (data not shown). On the other hand, the *RBCS1 *cDNA from *C. canephora *was strictly identical to the unigene SGN-U617577 (Figure 1). Altogether, these results confirmed those retrieved from the EST analysis, which demonstrated the existence of two homeologous genes of *RBCS1 *in *C. arabica*, one from the *C. canephora *sub-genome and another from the *C. eugenioides *sub-genome.

### Cloning of the *CcRBCS1 *gene

**Figure 2:**
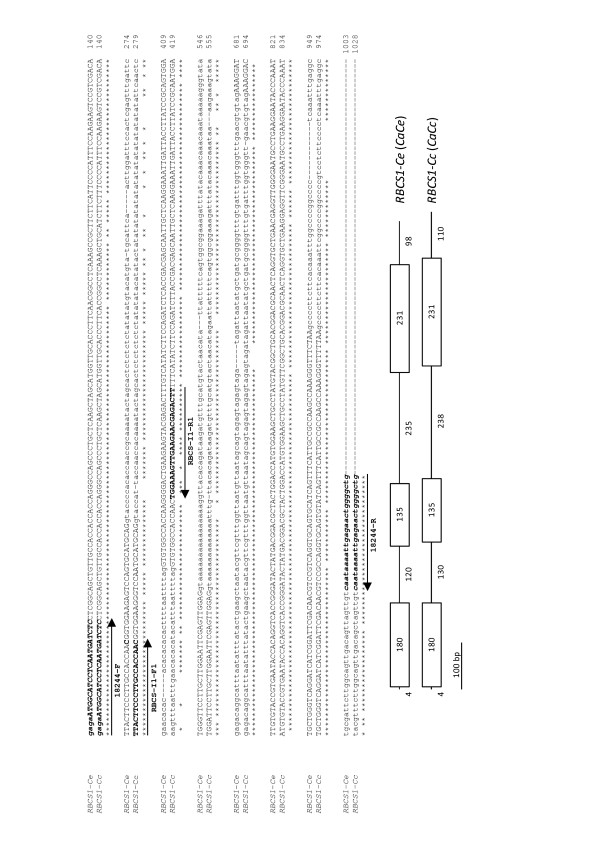
**Alignment of the *RBCS1 *genes from *C. arabica *and *C. canephora***. The *CaRBCS1 *gene [GenBank:AJ419827], previously cloned from *C. arabica *[58], corresponded to the *C. eugenioides *(*CaCe*: *RBCS1-Ce*) allele, while the *CcRBCS1 *gene [GenBank:FR772689, this work] corresponded to the *C. canephora *(*CaCc*: *RBCS1-Cc*) allele. Horizontal arrows as well as nucleotides are in bold and italics and correspond to primer sequences. The 18244-F and -R primers were used to amplify the *CcRBCS1 *(Table 1). The RBCS-I1-F1 (RBCS_intron1_F1) and -R1 (RBCS_intron1_R1) primers were used for the mapping of the *CcRBCS1 *gene [64]. The stars below the alignments indicate identical bases, and the nucleotides are numbered for each lane. A schematic representation of the *CaCe *and *CaCc *genes is also given. Exons are boxed and numbers indicate fragment sizes in base pairs.

The *RBCS1 *gene from *C. canephora *(called *RBCS1-Cc *or *CaCc*) was also cloned and sequenced (Figure 2). It shared 90% nucleotide identity with the *CaRBCS1 *gene from *C. arabica *that corresponds to the *RBCS1 *gene (called *RBCS1-Ce *or *CaCe*) of the *C. eugenioides *sub-genome. The two genes exhibited a similar structure and consisted of three exons and two introns. The sizes of the first and second introns were 120 bp and 235 bp for the *CaCe *allelic form and 130 bp and 238 bp for the *CaCc *allelic form, which therefore demonstrates inter-specific sequence polymorphisms. The nucleotide sequences differed by numerous single nucleotide polymorphisms (SNPs) and several insertion and deletion (indels) events in the introns and the 3' UTR region. Regarding the introns, it is worth noting that those of the *RBCS1-Cc *gene were always slightly longer than those of the *RBCS1-Ce *gene.

### The characteristics of the RBCS1 proteins

**Figure 3:**
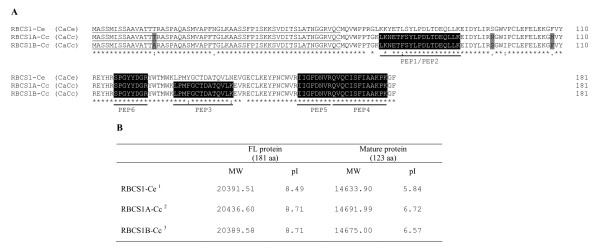
**Sequence alignment and characteristics of the coffee RBCS1 proteins**. (A): The amino acids corresponding to the chloroplastic transit peptide [1 to 58] are underlined. Identical amino acids are indicated by stars, conservative substitutions are indicated by two vertically stacked dots and semi-conservative substitutions are indicated by single dots. The RBCS1-Ce (CaCe) isoform from *C. eugenioides *corresponded to the proteins with the GenBank accession numbers CAD11990 and CAD11991 translated from the *CaRBCS1 *cDNA [GenBank:AJ419826] and gene [GenBank:AJ419827], respectively. The RBCS1A-Cc (CaCc) protein from the *CcRBCS1 *cDNA (FR728242) and gene (FR772689) sequences of *C. canephora *(this study) was strictly identical to the protein deduced from the SGN-U617577 unigene. The RBCS1B-Cc (CaCc) protein was deduced from the SGN-U607190 unigene. Divergent amino acids between RBCS1-Ce (CaCe) and RBCS1A-Cc (CaCc) proteins are boxed in grey, and those confirmed by mass spectrometry analysis (Table 6) are boxed in black. (B) The RBCS1-Ce (CaCe) protein deduced from the *CaRBCS1 *cDNA and gene sequences was identical to the protein deduced from the SGN-U607188 unigene (^1^). The RBCS1A-Cc protein was deduced from the *RBCS1-Cc *(identical to SGN-U617577^2^) cDNA and gene sequences from *C. canephora *(this study). The RBCS1B-Cc protein was deduced from the SGN-U607190 (^3^) nucleic acid sequence. Molecular weights (MW in Daltons), amino acids (aa) and isoelectric points (pI) are indicated for full-length (FL) and mature (without the chloroplast transit peptide) RBCS1 proteins. SGN sequences were obtained from the Sol Genomics Network http://solgenomics.net/content/coffee.pl.

An *in silico *analysis of these sequences was performed to define the characteristics of the corresponding RBCS1 proteins. All of them contained a 543-bp open reading frame coding for a protein of 181 amino acids (Figure 3A). The RBCS1-Ce (CaCe) protein was deduced from the unigene SGN-U607188 from *C. arabica *and was identical to that deduced from the *CaRBCS1 *cDNA and gene sequences. The protein has a theoretical molecular mass of 20391 Da and an estimated isoelectric point (pI) of 8.49 (Figure 3B). By homology with other chloroplastic proteins encoded in the nucleus [59], the first 58 amino acids corresponded to a putative chloroplast transit peptide. Consequently, the theoretical molecular mass of the mature RBCS1-Ce should be 14633 Da with a pI of 5.84. On the other hand, two isoforms of the RBCS1-Cc protein could be deduced from the nucleic sequences of *C. canephora*: RBCS1A-Cc coded by the *RBCS1-Cc *cDNA (this study) and RBCS1B-Cc deduced from the SGN-U607190 unigene. In their mature forms, the RBCS1A-Cc and RBCS1B-Cc proteins should have a molecular mass of 14691 and 14675 Da and estimated pIs of 6.72 and 6.57, respectively. This analysis suggests that different RBCS1 isoforms exist and are characterised by similar molecular weights but differing theoretical pIs.

### *RBCS1 *gene expression in different genotypes and species of *Coffea*

According to the sequence alignments, primer pairs specific for each of the *RBCS1 *homeologous genes (*CaCc = RBCS1-Cc *and *CaCe *= *RBCS1-Ce*) were designed (Table 1) and quantitative PCR assays were performed to analyse *RBCS1 *expression in leaves of coffee plants from different species and genotypes by measuring the *CaCc *and *CaCe *expression levels (Table 2). From a technical point of view, cross-hybridisation of primers against the two different *RBCS1 *genes was excluded because the melting curves clearly separated the *CaCc *and *CaCe *amplicons produced using the C18244 and E18244 specific primer pairs, respectively (data not shown). Using the C18244 primer pair, high expression of the *CaCc *homeologous gene was observed in leaves of Conilon clones of *C. canephora*. On the other hand, *CaCc *was weakly expressed in leaves of *C. arabica *genotypes, particularly for those that did not undergo recent introgression with *C. canephora *genomic DNA, such as Typica, Bourbon, Caturra, Catuaí and Rubi, for example. The opposite situation was observed with the primer pair E18244, specific for the *RBCS1-Ce *(*CaCe*) haplotype from the *C. eugenioides *sub-genome of *C. arabica*. For *C. eugenioides*, the *CaCc*/*CaCe *expression was extremely low, which validates that there is almost an exclusive expression of the *CaCe *isoform in this species. Altogether, these results showed that *CaCe *and *CaCc *expression could be considered as negligible in *C. canephora *(high *CaCc*/*CaCe *ratio) and *C. eugenioides *(low *CaCc*/*CaCe *ratio), respectively. The results also demonstrated a large variability of *CaCc *expression in leaves of the two studied Timor hybrids. Both *CaCc *and *CaCe *homeologous genes were expressed to similar levels (*CaCc*/*CaCe *= 0.4) in the HT832/2 genotype, whereas *CaCc *expression was undetected (*CaCc*/*CaCe *= 4.10^-5^) in HT832/1 (Table 2). In introgressed *C. arabica *genotypes coming from breeding programs that used either HT832/2 or controlled crosses with *C. canephora*, a great variability in *CaCc/CaCe *ratios was also observed. For example, high *CaCc *expression was detected in leaves of the HT832/2-derived Obatã, Tupi, IAPAR59 (I59), IPR97 and IPR98 cultivars as well as in those of the interspecific controlled cross Icatú. However, *CaCc *gene expression was low in the HT832/2-derived IPR107 and Icatú-derived IPR102 and IPR106 genotypes. For all coffee genotypes analysed, levels of the total *RBCS1 *gene expression evaluated by the T18244 primer pair appeared quite similar (data not shown).

**Table 1:** List of primers used for gene cloning and quantitative PCR experiments

Gene name	Source gene	Primer name	Primer sequence	bp
UBI *	SGN-U637098	BUBI-F BUBI-R	5' AAGACAGCTTCAACAGAGTACAGCAT 3' 5' GGCAGGACCTTGGCTGACTATA 3'	104
GAPDH *	SGN-U637469	GAPDH-F GAPDH-R	5' TTGAAGGGCGGTGCAAA 3' 5' AACATGGGTGCATCCTTGCT 3'	59
*RBCS1-Cc *(*CaCc*)	SGN-U617577 FR728242	C18244-F C18244-R	5' CCGTCCTCTTCCCCTCAAAT 3' 5' CCTGAAAGTACAGCCCCAGTTC 3'	91
*RBCS1-Ce *(*CaCe*)	SGN-U607188 AJ419826	E18244-F E18244-R	5' TTGGCCCCGGCCCCTCAAATT 3' 5' CAGCTAAAAGTACAGCCCCAGTTC 3'	93
*RBCS1-T*		T18244-F T18244-R	5' CTAGCATGGTTGCACCCTTCA 3' 5' AGTAATGTCGACGGACTTCTTGGA 3'	77
*RBCS1-DNA*		18244-F 18244-R	5' GAGAATGGCATCCTCAATGATCTC 3' 5' CAGCCCCAGTTCTCAATTTTATTG 3'	660(C) 648(E)

Primers were designed using Primer Express software (Applied Biosystems). The source gene indicates the accession numbers of coffee cDNA and gene sequences found in the GenBank and SOL Genomics Network (SGN, http://solgenomics.net/content/coffee.pl[56]) libraries and used to design the primer pairs. The size of the amplicon is indicated in base pairs (bp). E: *C. eugenioides *corresponding to the *CaCe *(*RBCS1-Ce *isoform). C: *C. canephora *corresponding to the *CaCc *(*RBCS1-Cc *isoform). The *RBCS1-T *primer pair was used to amplify total-*RBCS1 *(*CaCe*+*CaCc*) transcripts. The *RBCS1-DNA *primer pair was used to amplify the *CaCc *cDNA and gene sequences. Primer sequences of reference genes previously reported by Barsalobres-Cavallari *et al. *[101] are also given (*).

**Table 2:** The expression of *RBCS1 *isoforms in leaves of different coffee genotypes.

Genotype	Cultivar	Origin	Trial	*CaCc*/*CaCe*
*C. canephora*				

	L21		I	65.93
	14 ^T^	Conilon	G	1324.28
	22 ^S^	Conilon	G	247,10
	73 ^T^	Conilon	G	260.65
	120 ^T^	Conilon	G	236.71

*C. arabica *("pure")				

	Rubi ^S^	Mundo Novo x Catuaí	E	0.00013
	Bourbon		I	0.00014
	Typica		I	0.00017
	Catuaí	Mundo Novo x Caturra	I	0.00021

*C. arabica *("introgressed")				

	HT832/1	Timor hybrid	E	0.00004
	HT832/2	Timor hybrid	I	0.40102
	Icatú	*C. canephora *x Bourbon	I	9.33
	IAPAR59 ^T^	Villa Sarchi x HT832/2 (Sarchimor)	I	3.22
	Tupi	Villa Sarchi x HT832/2 (Sarchimor)	I	2.63
	Obabã	[Villa Sarchi x HT832/2] x Catuaí	I	1.26
	IPR97	Sarchimor	I	4.98
	IPR98	Sarchimor	I	21.65
	IPR102	Icatú x Catuaí	I	0.00427
	IPR106	Icatú x Catuaí	E	0.03212
	IPR107	Sarchimor x Mundo Novo	E	0.12255

*C. eugenioides*			I	0.00035

Expression was measured by the ratio *CaCc*/*CaCe *where *CaCc *(*RBCS1-Cc*) and *CaCe *(*RBCS1-Ce*) values were obtained using the C18244 and E18244 primer pairs (Table 1), respectively. Relative quantifications (RQ) were normalised using the expression of the *CcUBQ10 *(in the case of *C. canephora*) or *GAPDH *(for other species) reference genes. The *CaCc*/*CaCe *ratio corresponded to (1+E)^-ΔCt^, where ΔCt = Ct_mean_*CaCc *- Ct_mean_*CaCe *with E as the efficiency of the gene amplification. Leaves were collected from plants grown in the field at the Embrapa Cerrados (E), IAPAR station (I) and UFV greenhouse (G). When known, the reaction to drought is indicated (^T ^= Tolerant and ^S ^= Susceptible). All Sarchimors are derived from HT832/2.

### *RBCS1 *gene expression in leaves of *C. canephora *subjected to water stress

**Figure 4:**
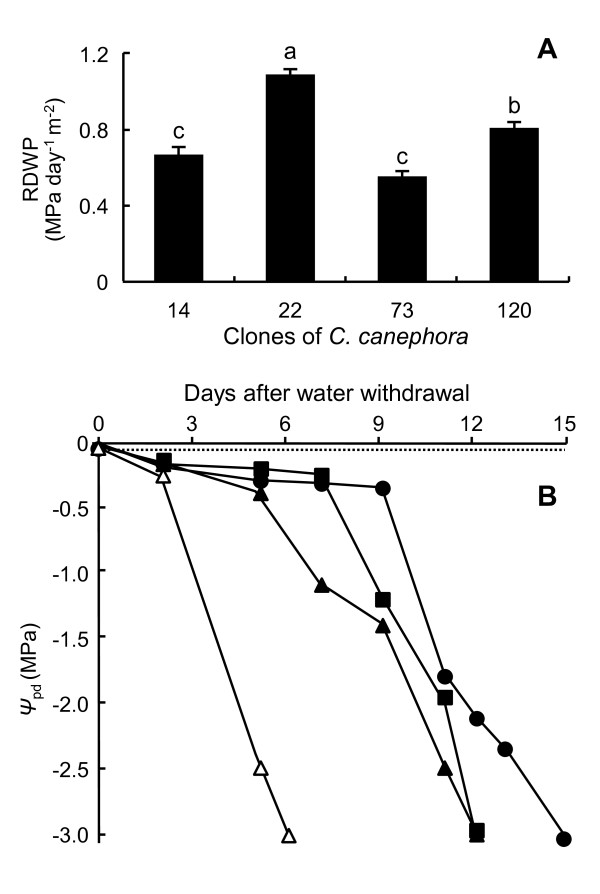
**The evolution of predawn leaf water potentials (*Ψ*_pd_) in the leaves of *C. canephora***. The clones 14, 22, 73 and 12 of *C. canephora *var. Conilon were grown in a greenhouse under water stress. The rate of decrease of *Ψ*_pd _(RDPWP) is indicated for each clone without irrigation (NI) in MPa day^-1 ^m^-2 ^(A). Different small letters represent significant differences between means for drought-stressed clones by the Newman-Keuls test at P ≤ 0.05 (clone effect). Values are means ± SD of three replicates. (B) For each clone, *Ψ*_pd _evolutions are presented relative to the days after water withdrawal (Δ, clone 22-NI; ▲, 14-NI; ■, 120-NI and ●, 73-NI).

The rate of decrease in the predawn leaf water potential (*Ψ*_pd_) (RDPWP) is one of the physiological parameters that distinguished the drought-susceptible clone 22 of *C. canephora *var. Conilon from the drought-tolerant clones 14, 73 and 120 [60,61]. To reach the imposed *Ψ*_pd _of -3.0 MPa for the stressed (NI) condition in the greenhouse, the RDPWP decreased faster for the clone 22 than for drought-tolerant clones (Figure 4A). In this condition, the clones 22 reached the *Ψ*_pd _of -3.0 MPa within six days, while clones 14, 73 and 120 reached the same within 12, 15 and 12 days, respectively (Figure 4B). As a control and for all the clones, the *Ψ*_pd _values of plants under irrigation were close to zero, which confirms the unstressed condition.

**Figure 5:**
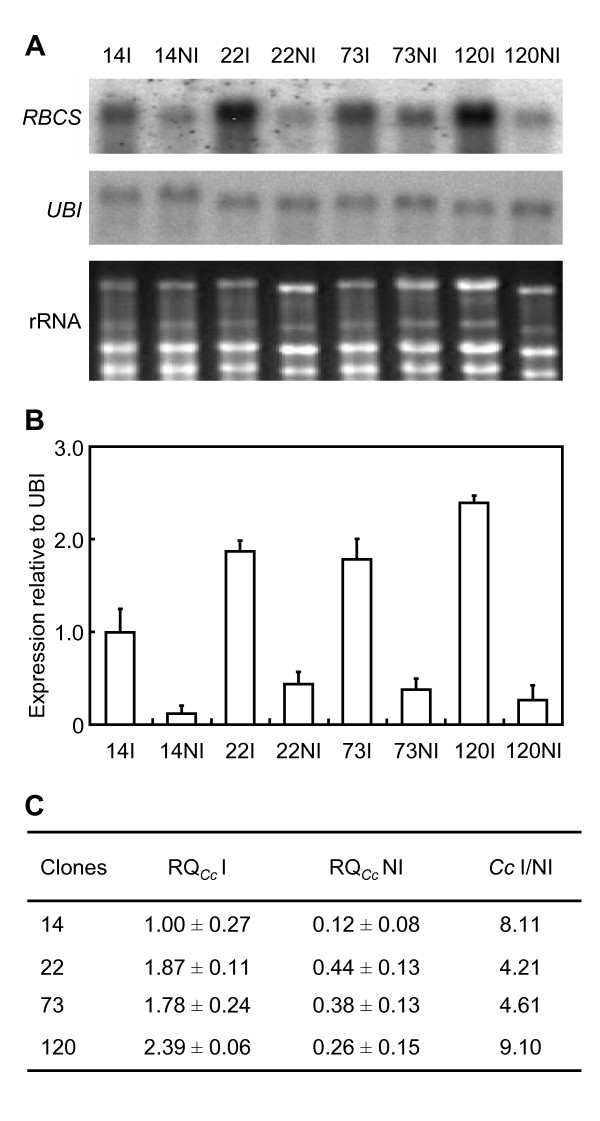
**The expression profiles of *RBCS1 *in *C. canephora***. For northern blot experiment (A), total RNAs (15 μg) were extracted from leaves of clones 14, 22, 73 and 120 of Conilon grown with (I) or without (NI) irrigation, separated by agarose gel electrophoresis and hybridised independently with *CcRBCS1 *(*RBCS*) and *CcUBQ10 *(*UBI*) cDNA probes. Total RNA (rRNA) stained with ethidium bromide was used to monitor equal loading of the samples. (B) The qPCR analysis was performed using the C18244 primer pair specific for the *CaCc *isoform of the *RBCS1 *genes. Expression levels are indicated in relative quantification of *RBCS1 *transcripts using the expression of the *CcUBQ10 *gene as a reference. Results are expressed using 14I as an internal calibrator. In each case, values are the mean of three estimations ± SD. (C) Values of relative quantification (RQ) are given for clones 14, 22, 73 and 120 grown with (I, *Ψ*_pd _≈ -0.02 MPa) or without (NI, *Ψ*_pd _≈ -3.0 MPa) irrigation. *RBCS1 *targets correspond to the *CaCc *gene amplified with the C18244 primer pair. The I/NI ratio of *RBCS1-Cc *gene expression (*Cc *I/NI) is also indicated.

The effects of WS on *RBCS1 *gene expression were analysed in leaves of these clones grown under I and NI conditions by a northern blot experiment with an internal *RBCS1 *cDNA fragment as a probe (Figure 5A). For all the clones, *RBCS *transcripts of the expected size (approx. 0.9 kb) were highly detected under the irrigated condition and poorly accumulated under WS. As an internal control, the expression of the *CcUBQ10 *(ubiquitin) reference gene appeared equal for all samples. The expression of *RBCS1 *alleles was also studied by quantitative PCR (qPCR) for the same clones using the expression of the *CcUBQ10 *gene as an internal reference (Figure 5B). For all clones, the *CaCe *expression was negligible, and relative quantification of *CaCc *(RQ*_Cc_*) was chosen to reflect total *RBCS1 *expression (Figure 5C). This analysis also confirmed reduction of *CaCc *gene expression (*CaCc *I/NI ranging from 4- to 9-fold) with WS. In addition, some differences in *RBCS1 *expression were observed between the clones but they were not correlated with phenotypic sensitivity to drought. Identical qPCR results were also obtained using *GAPDH *as a reference gene (data not shown).

### *RBCS1 *gene expression in leaves of young plants of *C. arabica *subjected to water stress

The effects of WS on *RBCS1 *gene expression were further analysed in leaves of young plants of Rubi and introgressed I59 cultivars grown in field conditions with (I) or without (NI) irrigation during two consecutive years (2008 and 2009). Two points of analysis were performed every year. The unstressed condition (U) corresponded to the rainy periods and the water stress (WS) condition to the dry season (Table 3). In this case, drought was not imposed but determined by the natural rainfall pattern during the dry-wet season cycle. For both cultivars, *Ψ*_pd _values of irrigated plants, during the dry season, ranged from -0.11 to -0.38 MPa, demonstrating the absence of drought stress. For the NI treatment, lower (more negative) values of *Ψ*_pd _were observed in 2008 than in 2009, demonstrating that the dry season was more severe during the former than in the latter. In addition, *Ψ*_pd _values measured during the dry season of 2008 and 2009 were almost less negative for the cultivar I59 than for Rubi, indicating a better access to soil water for I59 than for the Rubi cultivar.

**Table 3:** Predawn leaf water potentials (*Ψ*_pd_) measured in field tests of *C. arabica*.

Cultivar	Y	Irrigated (I)			Non-Irrigated (NI)		
		U	WS		U	WS	
I59	2008	-0.23 ± 0.09	-0.38 ± 0.10		-0.21 ± 0.05	-0.80 ± 0.12	
Rubi	2008	-0.19 ± 0.02	-0.22 ± 0.07		-0.19 ± 0.06	-1.88 ± 0.26	
I59	2009	-0.06 ± 0.02	-0.12 ± 0.00		-0.07 ± 0.02	-0.59 ± 0.03	
Rubi	2009	-0.06 ± 0.02	-0.11 ± 0.00		-0.13 ± 0.04	-1.20 ± 0.16	
Icatú	2010	*nd*			<-4.0		
Rubi	2010	*nd*			<-4.0		
Obatã	2010	*nd*			<-4.0		
I59	2010	*nd*			<-4.0		

							

**Cultivar**	Y	**Irrigated (I)**			**Non-Irrigated (NI)**		
		**U1**	**WS**	**U2**	**U1**	**WS**	**U2**

I59	2008	-0.41 ± 0.03	-0.37 ± 0.05	-0.14 ± 0.03	-0.66 ± 0.03	-1.35 ± 0.09	-0.15 ± 0.03
Rubi	2008	-0.28 ± 0.05	-0.20 ± 0.04	-0.17 ± 0.04	-0.45 ± 0.04	-1.96 ± 0.13	-0.18 ± 0.03

Young (top) and adult (bottom) plants were grown under irrigated (I) or non-irrigated (NI) conditions. *Ψ*_pd _values are expressed in mega-Pascal (MPa) and standard deviations (n = 9 leaves) are also indicated. For young plants, *Ψ*_pd _was measured during the rainy season (U: unstressed) and the dry season (WS: water stress). For adult plants, *Ψ*_pd _were measured only during the dry season (WS) under irrigated (I) or with the irrigation suspended for 90 days (NI) conditions. The points U1, WS and U2 corresponded to measurements before, during and after the return of irrigation, respectively. *nd*: *Ψ*_pd _potentials were not determined but ranged from -0.1 to -0.2 MPa under irrigation. The year of analysis (Y) is also indicated.

Q-PCR reactions used the primer pairs E18244, C18244 and T18244 to detect *CaCe *(Ce), *CaCc *(Cc) and total-*RBCS1 *(RQ*_RBCS1-T_*) expression, respectively (Table 4). Independent of water conditions, expression of both the *CaCc *and *CaCe *homeologs was always detected in the I59 cultivar, whereas *CaCc *expression was not detected in Rubi. It is also worth noting that total *RBCS1 *was mostly higher in I59 than in Rubi. For both cultivars, levels of RQ*_RBCS1-T_*were quite similar during the unstressed (rainy) condition of the year 2008. In comparison to the irrigated (I) condition, RQ*_RBCS1-T_*was reduced by 30% and 90% in NI plants of the I59 cultivar in 2008 and 2009, respectively. In both cases, this reduction affected mainly *CaCc *expression. For the Rubi cultivar, the absence of irrigation (NI) also reduced total *RBCS1 *expression by more than 80% in 2008 and 2009.

**Table 4:** Daytime expression levels of *RBCS1 *genes in the leaves of young plants of *C. arabica*.

		Irrigated (I)				Non-Irrigated (NI)			
		
	S - Y	RQ*_Cc_*	RQ*_Ce_*	RQ*_RBCS1-T_*	*Cc*/*Ce*	RQ*_Cc_*	RQ*_Ce_*	RQ*_RBCS1-T_*	*Cc*/*Ce*
	U-08	8.70	0.05	11.93	189.44	(*)	(*)	(*)	(*)
I59^(1)^	WS-08	32.80	0.09	32.66	253.33	21.83	0.13	22.49	167.92
	U-09	22.27	3.28	23.01	6.79	23.69	0.32	23.28	74.05
	WS-09	10.80	0.66	12.33	16.36	3.06	-	3.61	*nd*

	U-08	-	15.08	13.95	-	(*)	(*)	(*)	(*)
Rubi^(1)^	WS-08	-	14.39	12.15	-	-	1.97	2.25	-
	U-09	-	9.78	7.58	-	-	5.20	5.65	-
	WS-09	-	13.40	13.27	-	-	2.37	2.64	-

Icatú^(2)^	WS-10	13.73	1.14	11.95	12.04	3.69	0.61	3.42	6.09
Rubi^(2)^	WS-10	-	8.95	10.02	-	-	1.14	1.15	-
Obatã^(2)^	WS-10	*nd*	*nd*	12.19	-	*nd*	*nd*	3.60	-
I59^(2)^	WS-10	*nd*	*nd*	26.15	-	*nd*	*nd*	17.05	-

Nine-month-old plants (^1^: at the date of the U-08 point of analysis) grown in field conditions were studied during two consecutive years (2008 and 2009). Twenty-month-old plants (^2^: at the date of the WS-10 point of analysis) were analysed only during the dry season of 2010. For each cultivar, the season (S) and the year (Y) of analysis are indicated: U (unstressed condition) corresponding to the rainy season and WS (water stress) to the dry season. Corresponding predawn leaf water potentials (*Ψ*_pd_) are given in Table 3. *RBCS1 *gene expression was expressed in relative quantification (RQ) for the I59 (IAPAR59), Rubi, Icatú and Obatã cultivars grown in the field with (I) or without (NI) irrigation. *RBCS1 *targets correspond to *CaCe *(*Ce*), *CaCc *(*Cc*) and total *RBCS1 *(*RBCS1-T*) transcripts amplified by the E18244, C18244 and T18244 primer pairs (Table 1), respectively. *Cc*/*Ce *values corresponded to RQ*_Cc_*/RQ*_Ce_*ratios. For the U-08 point of harvest (*), qPCR analyses were not performed for non-irrigated (NI) plants that were considered identical to irrigated (I) ones. For the Rubi cultivar, *CaCc *gene expression was not detected (-). In that case, *CaCe *expression (RQ*_Ce_*) and total *RBCS1 *gene expression (RQ*_RBCS1-T_*) was deduced from qPCR experiments that used the E18244 and T18244 primer pairs. For Obatã and I59 cultivars analysed in 2010, *CaCe *(*Ce*)- and *CaCc *(*Cc*)-specific expression was not determined (*nd*), and RQ*_RBCS1-T_*was deduced from qPCR experiments that used the T18244 primer pair. Results were normalised using the expression of the *GAPDH *reference gene.

*RBCS1 *expression was also studied in the young plants of the Icatú, Rubi, Obatã and I59 cultivars subjected (NI) or not subjected (I) to the severe WS that occurred during the dry season of 2010, as shown in the Table 3. In the irrigated condition, the I59 cultivar showed highest values of total *RBCS1 *expression, while *RBCS1 *expression in the Rubi, Icatú and Obatã cultivars was lower and more similar (Table 4). Under NI condition, RQ*_RBCS1-T_*decreased for all cultivars, highly (-90%) for Rubi and to a lower extent (-70%) for Icatú and Obatã. Finally, the I59 cultivar was the genotype with the lowest decrease in *RBCS1 *gene expression; the value of RQ*_RBCS1-T _*during the NI treatment was 65% of that observed under irrigation.

### *RBCS1 *gene expression in leaves of adult *C. arabica *plants subjected to water stress: the effects of time of day

The effects of harvest hour on *RBCS1 *leaf expression were also studied using adult (eight-year old) plants of the Rubi and I59 cultivars grown in the field under continuous irrigation condition (I) or subjected to 90 days of WS during the dry season of 2008 (NI). The points of analysis were before (U1, unstressed), during (WS, water stress) and after (U2, unstressed) the dry season. As in young plants, the *Ψ*_pd _values measured for the non-irrigated (NI) treatment during the WS period were less negative for I59 than for Rubi (Table 3). On the other hand, the *Ψ*_pd _values ranged from -0.14 to -0.41 MPa for the irrigated (I) treatment, demonstrating the absence of WS.

*CaCc *expression (RQ*_Cc_*) decreased during the transition from U1 to WS under I and NI conditions in the I59 leaves harvested in the daytime (Table 5). However, *CaCe *gene expression was stable in plants irrigated continuously but decreased with WS in the NI condition. For the Rubi cultivar, *CaCe *expression (RQ*_Ce_*) was relatively stable under irrigated conditions for all points of the analysis. However, total *RBCS1 *expression (RQ*_RBCS1-T_*corresponding to RQ*_Ce_*) decreased with WS under NI treatment. The comparison of total *RBCS1 *expression levels between the two cultivars revealed higher (from 2- to 5-fold) expression in I59 than in Rubi, with a predominant expression of the *CaCc *over the *CaCe *homeolog in the former. For both cultivars, total *RBCS1 *expression values were similar before (U1) and after (U2) the WS period, demonstrating gene expression recovery with the return of irrigation.

**Table 5:** The expression levels of *CaCc *and *CaCe *isoforms in leaves of eight-year-old *C. arabica*.

	WT	Y	RQ*_Cc_*	RQ*_Ce_*	RQ*_RBCS1-T_*	*Cc*/*Ce*
		U1-08	21.65	6.22	27.87	3.48
	I	WS-08	6.95	8.99	15.94	0.77
I59		U2-08	35.17	6.21	41.38	5.66
	
		U1-08	20.56	5.46	26.03	3.76
	NI	WS-08	11.74	1.60	13.34	7.33
		U2-08	24.19	9.35	33.54	2.59

		U1-08	-	11.58	11.58	-
	I	WS-08	-	10.38	10.38	-
Rubi		U2-08	-	8.00	8.00	-
	
		U1-08	-	15.99	15.99	-
	NI	WS-08	-	9.52	9.52	-
		U2-08	-	12.46	12.46	-

						

	**WT**	**Y**	**RQ*_Cc_***	**RQ*_Ce_***	**RQ*_RBCS1-T_***	***Cc*/*Ce***

		U1-08	14.62	7.18	21.80	2.03
	I	WS-08	19.55	6.04	25.59	3.23
I59		U2-08	25.90	11.81	37.72	2.19
	
	NI	U1-08	35.32	8.16	43.48	4.33
		WS-08	17.68	6.12	23.80	2.89
		U2-08	19.62	6.65	26.26	2.95

		U1-08	-	7.21	7.21	-
	I	WS-08	-	8.30	8.30	-
Rubi		U2-08	-	8.79	8.79	-
	
		U1-08	-	14.05	14.05	-
	NI	WS-08	-	7.16	7.16	-
		U2-08	-	8.12	8.12	-

Leaves of the cultivars I59 and Rubi were harvested in daytime (top: between 10:00 and noon) or night time (bottom: between 3:00 and 5:00 am). The points of analysis were before (U1, unstressed), during (WS, water stress) and after (U2, unstressed) the dry season of 2008. Corresponding predawn leaf water potentials (*Ψ*_pd_) are given in the Table 3. The results of the relative quantification (RQ) are given for the cultivars IAPAR59 (I59) and Rubi grown in the field with (I) or without (NI) irrigation during the dry season (WT: water treatment). *RBCS1 *targets corresponded to *CaCe *(*Ce*) and *CaCc *(*Cc*) amplified by the E18244 and C18244 primer pairs (Table 1), respectively. Total *RBCS1 *expression (RQ*_RBCS1-T_*) corresponded to RQ*_Cc_*+ RQ*_Ce_*, while *Cc*/*Ce *ratios corresponded to the RQ*_Cc _*/RQ*_Ce_*ratios. For the Rubi cultivar, *CaCc *gene expression was not detected (-). Results were normalised using the expression of the *GAPDH *reference gene.

*RBCS1 *expression was also analysed when measuring *Ψ*_pd _in leaves harvested at night (Table 5). As observed for daytime, total *RBCS1 *expression was higher in I59 than in Rubi. For the I59 cultivar, it is worth noting that total nocturnal *RBCS1 *expression during WS was higher than expression measured at daytime in the same plants. For Rubi, values of night-time *RBCS1 *expression were quite similar to those determined at daytime.

### Accumulation of RBCS protein in leaves of *C. canephora *subjected to water stress

**Figure 6:**
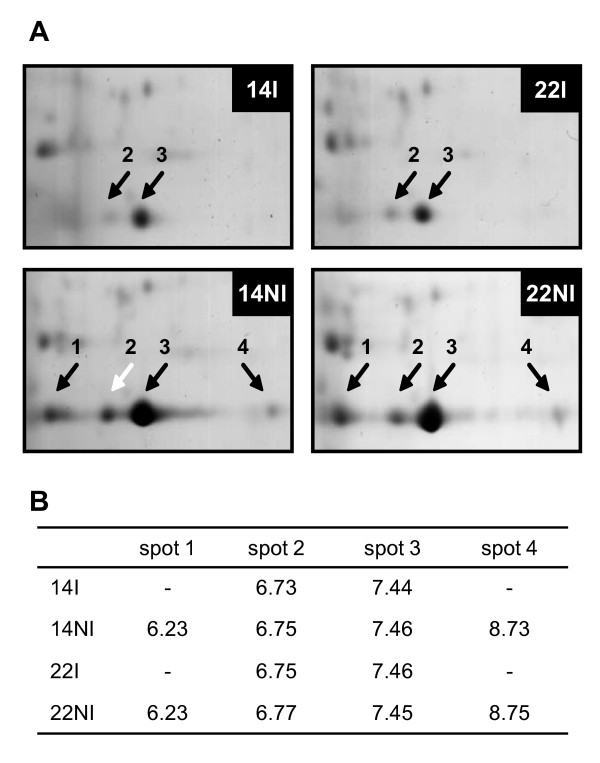
**Differential accumulation of RBCS subunits in leaves of *C. canephora *subjected to different water regimes**. A: Proteins were extracted from clones 14 and 22 grown with (I) or without (NI) irrigation (14I, 14NI, 22I and 22NI) and analysed by two-dimensional gel electrophoresis (2-DE). Only parts of the 2-DE gels containing RBCS proteins are shown. Black arrows indicate RBCS spots. The RBCS1 protein analysed by MS/MS is shown by a white arrow. B: Isoelectric points (pI) of RBCS proteins identified by 2-DE gel electrophoresis. The pIs of were determined from calibrated 2-DE gels using ImageMaster Platinum 6.0 Software. The absence (-) of a pI value indicates that the isoform was not present in the gel.

**Figure 7:**
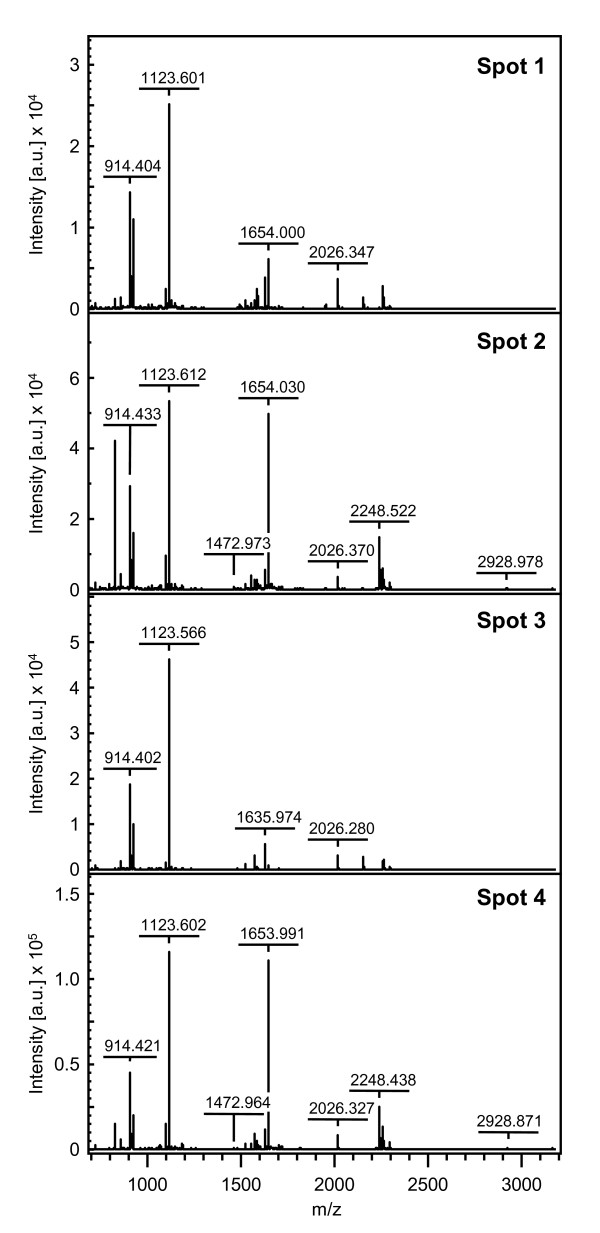
**Tryptic mass spectra of RBCS1 isoforms**. The spot numbers correspond to the RBCS1 isoforms identified by 2-DE gel electrophoresis in the leaves of clone 14 of *C. canephora *under the NI condition (see Figure 6). The x-axis represents the mass-to-charge (m/z) ratio and the y-axis represents the signal intensity of the ions expressed in arbitrary units (a.u.). For major peaks, corresponding monoisotopic m/z ratios are indicated.

**Figure 8:**
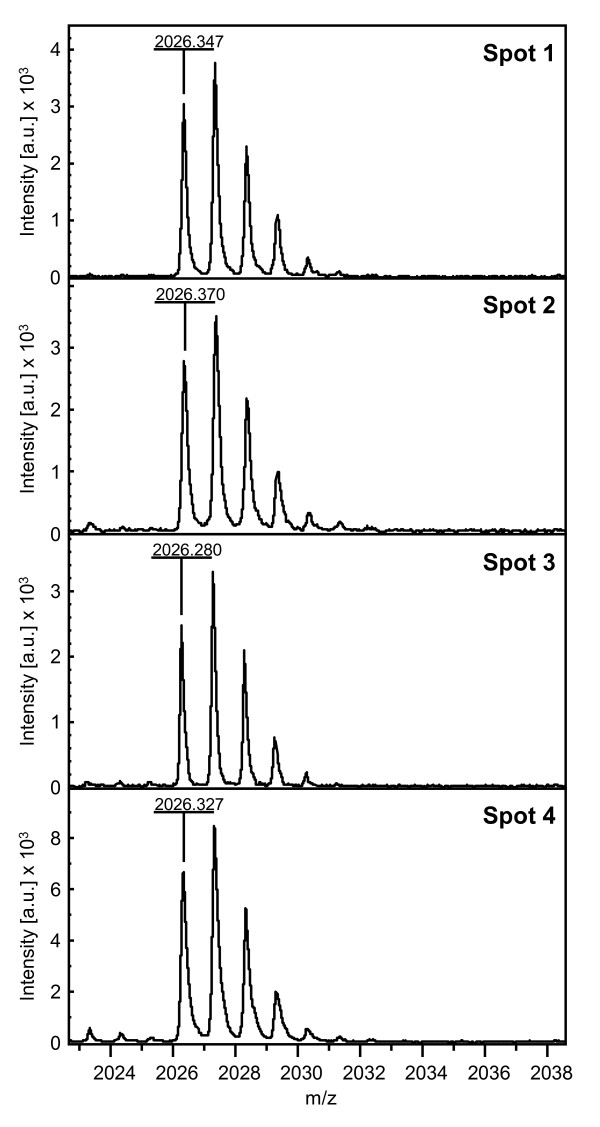
**Magnification of the tryptic mass spectra of peptide 2**. The spot numbers correspond to the RBCS1 isoforms identified by 2-DE in the leaves of clone 14 of *C. canephora *under the NI condition. The x-axis represents the mass-to-charge (m/z) ratio, and the y-axis represents the signal intensity of the ions expressed in arbitrary units (a.u.). The mass differences of the peaks correspond to the mass accuracy of MALDI-TOF/TOF.

**Figure 9:**
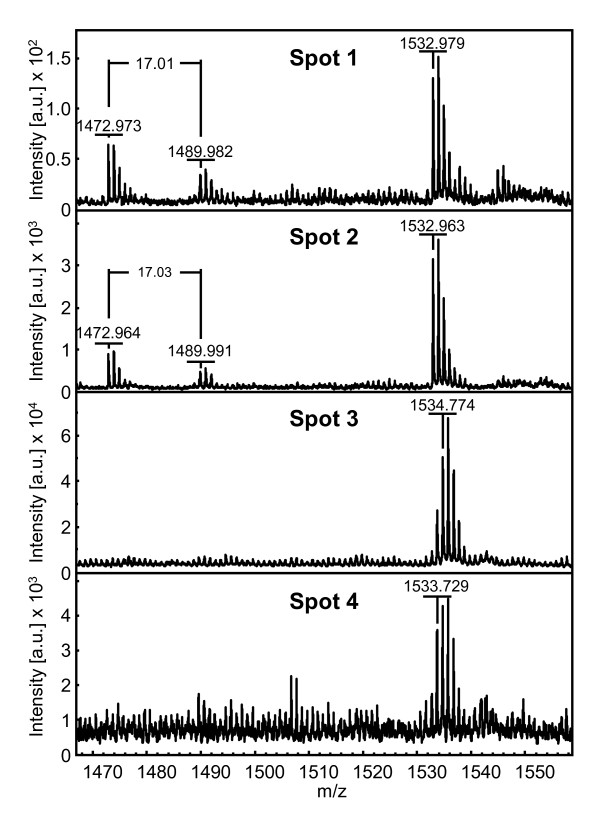
**Magnification of the tryptic mass spectra of the ions M+H 1472.9 and M+H 1489.9**. The spot numbers correspond to RBCS1 isoforms identified by 2-DE gel electrophoresis in the leaves of clone 14 of *C. canephora *under NI conditions. The x-axis represents the mass-to-charge (m/z) ratio and the y-axis represents the intensity of peaks expressed in arbitrary units (a.u.). The mass difference between ions of 17 Da corresponds to the loss of ammonia.

**Figure 10:**
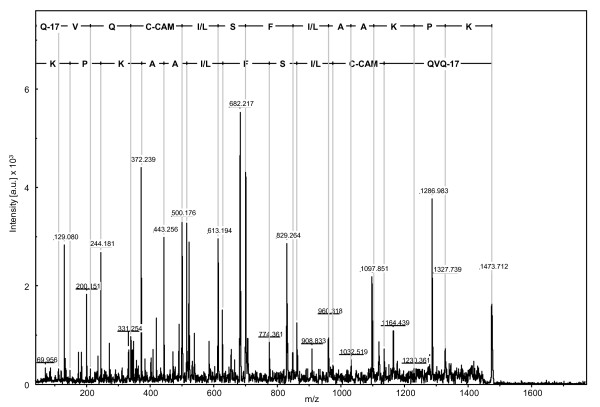
**MS/MS mass spectra of the ion M+H 1472.973 (peptide 4)**. This ion was isolated from RBCS1 spot 1 of clone 14 of *C. canephora *under NI conditions. The amino acid sequence of peptide 4 is indicated in the upper part of the graph. The x-axis represents the mass-to-charge (m/z) ratio, and the y-axis represents the intensity of peaks expressed in arbitrary units (a.u.).

**Figure 11:**
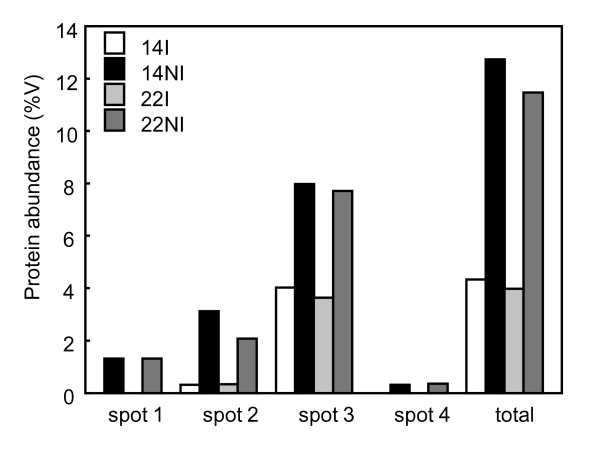
**Normalised protein abundance of RBCS1 isoforms**. Protein abundance was measured in clones 14 and 22 of *C. canephora *subjected (NI) or not subjected (I) to WS from the 2-DE and expressed in percentage volume (%V) of spots analysed by ImageMaster Platinum 6.0 software.

Soluble proteins were extracted from leaves harvested at night for clones 14 (drought tolerant) and 22 (drought susceptible) of *C. canephora *var. Conilon grown with (I) or without (NI) irrigation, and they were analysed by two-dimensional gel electrophoresis (2-DE). When looking at the gel portion containing the RBCS proteins, quantitative and qualitative changes of protein profiles were observed during WS (Figure 6A). For both cultivars, spots 2 and 3 were detected under the I and NI conditions. However, spots 1 and 4 were only detected under water stress. All were characterised by a similar molecular weight but differed in their pIs (Figure 6B). A detailed analysis of RBSC isoforms was performed for clone 14 under NI condition. Spot 2 (pI ≈ 6.7) was sequenced and resulted in six peptides (Table 6) that perfectly matched with the mature isoform of RBCS1 protein (Figure 3). Peptides 1 (M+H 2068.0) and 2 (M+H 2026.3) overlapped but differed in their N-terminal amino acid sequence by two residues. Peptides 4, 5 and 6 corresponded to the common regions of the CaCe and CaCc RBCS1 isoforms, while peptides 1, 2 and 3 matched only with the CaCc isoforms. For clone 14NI, the spectra of tryptic masses of spots 1 to 4 were very similar (Figure 7). Identical results were also obtained for spots 1 to 4 of clone 22NI (data not shown). In addition, peptide 2 corresponded to the ion M+H 2026.3 that was also observed in the spectra of all RBCS1 spots, which confirmed the similarity between these isoforms (Figure 8). For all of these isoforms, peptide mass fingerprinting of the tryptic digestion did not reveal post-translational modifications. This is justified by the fact that some tryptic peptides may not generally be represented in the mass spectrum, notably N-terminal peptides. Comparison of tryptic masses revealed that the ions with an m/z of 1472.9 and 1489.9, corresponding to peptide 4 (Figure 3A and Table 6), differed by 17 Da and characterised the loss of an ammonium group from the N-terminal sequence. They were present in RBCS1 spots 1 and 2 but absent in spots 3 and 4 (Figures 9 and 10). However, this peptide 4 was conserved in the CaCe and CaCc RBCS1 isoforms (Figure 3A). The normalised relative abundance, as evaluated by the percentage volume of the spots, clearly indicates an increase in all RBCS1 isoforms with drought stress (Figure 11). For example, the amount of RBCS spot 3 (pI ≈ 7.4) increased significantly under WS in the leaves of clones 14 and 22 (Figure 6A). However, quantitative differences between the two genotypes of *C. canephora *were not observed.

**Table 6:** Mass spectrometry analysis of the *RBCS1 *spot 2 isoform.

Peptides	Mass (M+H)	position	peptide sequences
1	2068.0015	68-86	LKNETFSYLPDLTDEQLLK
2	2026,3700	66-86	NETFSYLPDLTDEQLLK
3	1581.7596	130-143	LPMFGCTDATQVLK
4	1473.7050	167-179	QVQCISFIAAKPK
5	933.5152	159-166	IIGFDNVR
6	914.4002	116-123	SPGYYDGR

This spot was identified in the leaves of clone 14 of *C. canephora *under the WS condition. Six peptides from MALDI-TOF/TOF tryptic mass spectra (Figure 7) were sequenced by MS/MS ion search and *de novo *sequencing. They are also reported in Figure 3. The peptides position refers to the full-length RBCS1 protein. Peptide masses are shown as the monoisotopic mass (M+H).

## Discussion and conclusions

The mechanisms regulating Rubisco activity and its abundance during water stress (WS) are not well characterised. Some works have reported that the loss of Rubisco activity constitutes an early response to WS [37]. In contrast, there is also evidence that more severe stress or stress applied for a longer period also decreases the amount of Rubisco [62]. Numerous studies have investigated the expression of the *RBCS *genes in response to light or in different tissue types and have shown that transcripts from individual genes accumulate differently [for a review, see [63]]. In higher plants, the *RBCS *genes are very similar to each other, which results in only a few amino acid differences in the RBCS proteins. Considering that RBCS complements the structure of RBCL and that evolution is likely to have resulted in specialisation of the different RBCS proteins, it is possible that different RBCS genes may have different impacts on Rubisco activity and regulation [34]. In this context, the main aims of this work were to identify the alleles of coffee *RBCS1 *gene, to determine the expression of these genes in different species with an emphasis on the polyploid species *C. arabica *and to study the effects of WS on *RBCS1 *expression in different genotypes and environmental conditions.

The existence of homeologous coffee *RBCS1 *genes was revealed through a search of public databases of coffee ESTs homologous to the *CaRBCS1 *cDNA sequence previously cloned from *C. arabica *[53]. Here, we report the cloning and sequencing of the *RBCS1 *cDNA and its corresponding gene from *C. canephora *(*RBCS1-Cc*). Nucleic acid alignments demonstrated that the *RBCS1-Cc *cDNA matched with *RBCS1 *ESTs expressed in both the *C. canephora *and *C. arabica *cDNA libraries [55,56]. In the latter species, *RBCS1 *ESTs identical to the previously-cloned *CaRBCS1 *cDNA and gene sequences also corresponded to a *RBCS1 *read in *C. eugenioides*. *RBCS1 *sequence alignments also revealed the existence of some nucleic differences that could correspond to sequencing errors or real SNPs that characterise the different alleles of *RBCS1*. Access to coffee whole genome sequences could help to resolve these points [52]. It is also worth noting that all the *RBCS *genomic sequences amplified from the 12 different genotypes of *C. arabica *(L.S. Ramirez, unpublished results) were identical to *CaRBCS1 *rather than *RBCS1-Cc*. This should be explained by the fact that these genes were probably amplified by specific primers that recognise the *CaRBCS1 *allele carried by the *C. eugenioides *sub-genome. Altogether, these results clearly showed that two homeologous *RBCS1 *genes were expressed in *C. arabica*, one from the *CcRBCS1 *gene (also called *CaCc*), which was carried by the *C. canephora *sub-genome of *C. arabica*, and the other from the *CaRBCS1 *gene (also called *CaCe*), which was carried by the *C. eugenioides *sub-genome of *C. arabica*. Thus, our results once again confirmed that the ancient *C. canephora *and *C. eugenioides *genomes constitute the two different sub-genomes of *C. arabica *[3,4]. Comparison of the *RBCS1-Cc *and *RBCS1-Ce *(corresponding to *CaRBCS1*) gene sequences also revealed interspecific sequence polymorphisms characterised by several indels mainly in the introns and in the 3' UTR region. Intraspecific sequence polymorphisms were also observed in *C. canephora*, and they permitted the recent mapping of the *CcRBCS1 *gene to the G linkage group of the *C. canephora *genetic map [64].

The expression variability of *RBCS1 *alleles was further tested in different coffee species and genotypes of *C. arabica *using specific primer pairs designed to the 3' UTR region of the *RBCS1 *cDNAs. Our results clearly demonstrated high *CaCc *(with negligible expression of *CaCe*) expression in *C. canephora *and high *CaCe *(with negligible expression of *CaCc*) expression in *C. eugenioides*. After this validation, expression of the homeologous *RBCS1 *genes was analysed in the different genotypes of *C. arabica*. These highlighted the predominant expression of the homeologous *CaCe *over the *CaCc *genes in the leaves of non-introgressed ("pure") *C. arabica *cultivars such as Typica, Bourbon and Catuaí; the former two cultivars correspond to the base populations that generated the latter cultivar [65,66]. In a previous study, Petitot *et al. *[67] also reported that the *CaWRKY1a *and *CaWRKY1b *genes of *C. arabica*, which encode for transcription factors of the WRKY family, originated from the two parental sub-genomes of this coffee species. In that case, *CaWRKY1a *and *CaWRKY1b *were concomitantly expressed, and both homeologous genes contributed to the transcriptional expression of coffee defence responses to pathogens. The result presented here are quite different, because they clearly highlighted the predominant expression of the *CaCe *over the *CaCc *homeologous gene for the non-introgressed genotypes of *C. arabica *and suggested that specific suppression of *CaCc *expression occurred during the evolutionary processes that led to the creation of the *C. arabica *species. This observation is in agreement with the recent results of Vidal *et al. *[4], who reported that within *C. arabica*, the *C. eugenioides *sub-genome may express genes coding for proteins that assume basal biological processes (as is the case for photosynthesis), while the *C. canephora *sub-genome contributes to adjust *Arabica *gene expression by expressing genes coding for regulatory proteins.

Another noteworthy result concerned the differential expression of the *RBCS1 *homeolog genes in Timor hybrids HT832/1 and HT832/2 as well as in the Icatú- or HT832/2-derived (introgressed) varieties. The qPCR experiments presented here clearly showed that the *CaCe *and *CaCc *homeologs were co-expressed with the same order of magnitude in HT832/2, while *CaCc *expression was undetectable in HT832/1. Most of the HT832/2-derived cultivars showed preferential expression of *CaCc *over the *CaCe *homeolog. However, Icatú-derived IPR102 and IPR106, as well as HT832/2-derived IPR107, presented the inverse situation of low expression of *CaCc*. The simplest hypothesis would be the existence of one or several genetic factors activating the expression of sub-genome *CaCc *genes in the *C. canephora *(*Cc*) species when introgressed with *C. arabica*. The *RBCS1-Cc *gene itself might be this genetic factor, but it might also be one or several other introgressed genes involving epistatic regulation. Epistasis is now proven to have a crucial role in gene regulation [68] and even in the heterosis phenomena [69]. Under this hypothesis, pure *C. arabica *does not express *RBCS1-Cc *in the absence of the *Cc *genetic factor. Introgressed *C. arabica *does or does not include the *Cc *genetic factor depending on the actual *Cc *genome introgressed. During selection from introgressed material, the percentage of the *Cc *genome tends to decrease because i) backcrosses are often directed toward a pure *C. arabica *parent and ii) phenotypic selection is directed towards *C. arabica *characteristics. Indeed, only disease-resistant genes from *C. canephora *are desired, while other genes that are part of the genetic drag lead to a decrease in cup quality [70,71]. The recently introgressed *C. canephora *genome has been estimated to represent 8 to 27 percent of the whole genome of introgressed varieties of *C. arabica *[5]. However, Bertrand *et al *[71] showed that differences in the cup quality of various introgressed varieties was not explained by the quantity of the introgressed *C. canephora *genome, thus suggesting that the types of introgressed genes are more important than the quantity of introgressed genome. In our study, it could be hypothesised that the *Cc *genetic factor is absent in HT832/1 and present in HT832/2 accessions of Timor hybrids. HT832/2-derived introgressed lines express *RBCS1-Cc *if the *Cc *factor has been maintained in the selection process. This would be the case for all HT832/2-derived varieties except in IPR107, which might have lost the *Cc *genetic factor during selection. Under this hypothesis, both Icatú-derived genotypes IPR102 and IPR106 would have lost the *Cc *genetic factor. No HT832/1-derived varieties were part of our study. However, such varieties should not express *RBCS1-Cc*, as the *Cc *factor would be absent in HT832/1. Checking the expression of *RBCS1-Cc *in HT832/1-derived varieties would thus reinforce or discard the hypothesis of a *Cc *genetic factor that epistatically regulates the expression of this gene. However, *RBCS1 *represents a potentially useful model to explore the differential expression of both the *CaCc *and *CaCe *sub-genome of *C. arabica*. Such experiments would advance our understanding of how epistasis regulates the gene expression of different sub-genomes in an amphidiploid species. Differential gene expression from both whole sub-genomes of *C. arabica *has been recently studied through a coffee-specific microarray [72]. This work allowed a more specific study that showed a preferential general expression of *CaCc *and *CaCe *genes at higher and lower temperatures, respectively [73]. Well-designed *RBCS1 *expression studies might provide a powerful single gene model for drought resistance and epistatic regulation in an amphidiploid and may contribute to a better understanding of epigenetic regulation in plant polyploids and its relationship to polyploidy advantages [74]. Recent genomic resources from *C. canephora*, including a dense genetic map [64], will help precise tracking of the introgressed *C. canephora *genome and possibly aid in understanding the functioning of the *Cc *genetic factor responsible for *CaCc *expression in introgressed varieties.

Another aim of this work was to study the effects of WS on *RBCS *expression in different coffee species. In higher plants, several works reported the rapid decrease in abundance of *RBCS *transcripts with WS and, consequentially, a reduction in RBCS protein accumulation in leaves [30-34,75]. In our conditions, it is worth noting that the decreases of *Ψ*_pd _were much slower for field-grown plants of *C. arabica *than those for *C. canephora *grown in a greenhouse. In addition, and except in 2010, the *Ψ*_pd _values observed for *C. arabica *during the period of maximum WS, were much less negative than those of *C. canephora*. This clearly demonstrated that the WS conditions were not equivalent between the two studies and that the stress suffered by the clones of *C. canephora *was more severe than the stress applied to the Rubi and I59 cultivars of *C. arabica*. For the latter and in young and adult plants, *Ψ*_pd _values under WS always appeared less negative for I59 than Rubi indicating better access to soil water for the former than the latter [76]. Regarding *RBCS1 *gene expression, the results presented here clearly showed a drastic decrease in total *RBCS1 *transcripts with severe WS for *C. canephora*. Irrespective of the clone analysed, total *RBCS1 *(*CaCc*) gene expression was reduced by 75% in WS-plants with a leaf predawn water potential (*Ψ*_pd_) of -3.0 MPa. Independently of plant age, a drought-induced decrease of total (daytime) *RBCS1 *transcripts was also observed for the two field-grown cultivars (Rubi and I59) of *C. arabica *subjected to WS. For I59, WS reduced the daytime expression of both the *CaCc *and *CaCe *homeologous genes, whereas only *CaCc *expression declined at night. Q-PCR experiments showed higher *RBCS1 *gene expression in I59 but also showed a lower extent of gene expression for the Icatú and Obatã cultivars than for the Rubi cultivar. For the latter, total *RBCS1 *gene expression was lower at night time than at daytime suggesting reduced transcription or an increase in transcript turnover under nocturnal conditions. However, the opposite seems to occur for the I59 cultivar, which shows a nocturnal increase in total *RBCS1 *gene expression, mainly mediated by enhanced *CaCc *expression. Together with the *Ψ*_pd _measurements, these results demonstrate the different behaviours of *C. arabica *cultivars during drought stress and suggest that those introgressed with a *C. canephora *genome could better tolerate WS conditions than cultivars of "pure" *C. arabica*.

It is well known that expression of the *RBCS *genes is positively regulated by light [for a review, see [77]]. In addition, several works also reported that increased sugar (*e.g*., glucose and fructose) levels can trigger repression of photosynthetic gene transcription including *RBCS *[24]. However, diurnal *RBCS *expression and light/dark oscillation of *RBCS *mRNA in an inverse timeframe to the normal daytime accumulation and night mobilisation of leaf carbohydrates previously reported [78-81]. Praxedes *et al. *[82] showed increased concentration of sucrose and hexoses, probably coming from enhanced starch degradation, in WS leaves of clone 120 of *C. canephora *that could also be explained by the daytime decrease of *RBCS1 *transcripts reported here.

In order to see if this reduction in *RBCS1 *gene expression also affected the amount of the corresponding protein, 2-DE experiments were performed to study RBCS1 proteins in the leaves of clones 14 (drought-tolerant) and 22 (drought-susceptible) of *C. canephora *var. Conilon grown with (I) or without (NI) WS. For both clones, drought stress increased the amount of the main RBCS1 isoform corresponding to spot 3 and also led to the accumulation of at least three other RBCS isoforms of identical molecular weight but different pIs. Comparison of the tryptic mass profile by peptide mass fingerprinting revealed the absence of some peptides in the different RBSC1 isoforms, such as for spots 3 and 4, that did not contain peptide 4. In addition, other ions that could correspond to this peptide were not found. It is possible that peptide 4 was not detected due to posttranslational modifications that modify its mass. Another possibility is that spots 3 and 4 really corresponded to RBCS alleles that differed from RBCS1 proteins as in *C. arabica*, where differential expression of RBSC alleles under drought stress was observed (Ramos, personal communication). In the literature, few examples showed up-regulation of *RBCS *gene expression with drought stress accompanied by the Rubisco increase [83-85]. Altogether, the results presented here suggest a decoupling between *RBCS1 *gene expression and the accumulation of RBCS1 protein during WS.

Several hypotheses could be proposed to explain why the decline of photosynthetic CO_2 _fixation (*A*) with drought stress previously reported for clones 14 and 120 of *C. canephora *var. Conilon [60] is not accompanied by a decrease in amount of RBCS1 protein. The first hypothesis could involve the participation of Rubisco binding proteins (RBP) that stabilise, protect and activate the Rubisco holoenzyme under adverse environmental conditions [86]. Proteins such as chaperones, Rubisco activase, Clp ATP-dependent calpain protease and detoxifying enzymes have been shown to play such roles that favour Rubisco accumulation and stabilisation by preventing its damage under drought stress [26,41]. It is worth noting that WS increased expression of genes coding for small HSP proteins, as observed in the leaves of clones 14 and 22 of *C. canephora *[87]. In addition, high activities of detoxifying enzymes (*e.g.*, ascorbate peroxidase and superoxide dismutase) were also reported in the leaves of water-stressed clones 14 and 120 of *C. canephora *[47]. The second possibility is that accumulation of RBCS1 protein could come from the expression of other *RBCS *alleles up-regulated during WS to compensate for the down-regulation of *RBCS1*. However, because the decrease of *RBCS1 *gene expression was confirmed by qPCR experiments using different primer sets, including one pair designed to the RBCS-coding sequence that should be extremely conserved within the coffee RBCS gene family, this hypothesis seems unlikely. A third possibility is that RBCS1 protein accumulated under WS came from the translation of *RBCS1 *mRNA transcribed overnight. This hypothesis cannot be completely ruled out because nocturnal *RBCS1 *expression was effectively observed in leaves of the I59 and Rubi cultivars of *C. arabica*. In that case, nocturnal accumulation of *RBCS1 *mRNAs could participate in maintaining the high daytime amount of RBCS1 protein even under a sharp reduction in *RBCS1 *gene expression. This should also favour a quick recovery of photosynthetic capacity under favourable environmental conditions and help coffee plants to cope with WS [38].

Water stress can directly affect photosynthesis by causing changes in plant metabolism or by limiting the amount of CO_2 _available for fixation [35]. Although stomatal closure generally occurs when plants are exposed to drought, in some cases photosynthesis may be more controlled by the capacity to fix CO_2 _than by increased diffusive resistance [88]. If Rubisco is not a limiting enzyme for carbon fixation under drought, the impaired activity of enzymes involved in the regeneration of Rubisco or in the Calvin cycle (*e.g*., sedoheptulose-1,7-bisphosphatase and transketolase) could be responsible for the drought-induced decrease in photosynthetic capacity [89]. In addition to the carboxylase activity, Rubisco has also oxygenase activity. This process, called photorespiration, can protect the photosynthetic apparatus against photoinhibition by keeping the electron transport chain active, thus limiting electron accumulation and ROS formation. This could explain why photoinhibitory damages were not observed in water-stressed coffee plants [47,60,90]. In that case, Rubisco could confer acclimatisation to oxidative stress under water deficit. The true mechanism of Rubisco contribution to water and oxidative stress responses in coffee plants still remains obscure and highlights the necessity for additional detailed studies to precisely determine its contribution.

The work presented here is the first to (a) investigate the effects of drought stress on gene expression with coffee plants grown in the field, (b) compare these results with those obtained for coffee plants grown under WS intensities and (c) analyse the transcriptome response of the two main coffee species, while taking into account the complex regulation of homeolog genes in *C. arabica *and opening the way to further sharpen understanding of epistatic regulation of sub-genome expression in an amphidiploid species. These results constitute only one part of a broader project that aims to study the effects of drought stress on biomass, architecture, anatomy and eco-physiological parameters of Rubi and I59 cultivars [61,91]. The integration of these data with ongoing studies of candidate genes should help us to understand the genetic determinants of drought tolerance in coffee, which constitutes an essential step in the improvement of coffee-breeding programs.

## Methods

### Plant material

Different plant material was used in this study depending on the specific experiments. So-called Timor Hybrids were not first-generation crosses but rather originated from various backcrosses with *C. arabica *after an initial cross [92]. The main three Timor Hybrids (HT832/1, HT832/2 and HT1343) were used in *C. arabica *breeding programs [5,93]. In our study, all Timor Hybrid introgressed varieties were derived from HT832/2. Controlled crosses also led to the Icatú F1 cross between *C. canephora *and *C. arabica *[94]. After backcrosses with *C. arabica*, Icatú-derived varieties were selected. In summary, we used the following material (Table 2):

• two diploid species: *C. canephora *(*Cc*) and *C. eugenioides *(*Ce*)

• four varieties of *C. arabica *amphidiploid species whose sub-genomes are related to present *C. canephora *(*CaCc*) and *C. eugenioides *(*CaCe*)

• one controlled F1 cross between *C. canephora *and *C. arabica*: Icatú

• two natural *C. arabica *introgressed hybrids: HT832/1 and HT832/2

• various introgressed HT832/2- or Icatú-derived varieties

#### Evaluation of RBCS1 gene expression in different genotypes of C. arabica

The plants of the genotypes Tupi, Bourbon, Typica, Catuaí, HT832/1, HT832/2 and IPR (97 to 107 [93]) from *C. arabica *as well as plants of *C. eugenioides *and *C. canephora *(clone L21) were cultivated on the coffee collection of the IAPAR (Instituto Agronômico do Paraná, Londrina, Brazil 23°21'17"S - 51°10'00"W) experimental station without WS (Table 2).

#### The effects of water stress on RBCS1 gene expression in C. canephora

Drought stress experiments used *C. canephora *clones (drought-tolerant: 14, 74 and 120; drought-susceptible 22) of the Conilon variety previously identified by the Incaper (Instituto Capixaba de Pesquisa, Assistência Técnica e Extensão Rural, Espírito Santo, Brazil). Rooted stem cuttings were grown in greenhouse conditions (UFV- Universidade Federal de Viçosa, Minas Gerais, Brazil) in small (12 l) containers [95]. When the plants were 6 months old, water deficit was imposed by withholding watering to reach a predawn leaf water potential (*Ψ*_pd_) of around -3.0 MPa for WS condition (Figure 4).

#### The effects of water stress on RBCS1 gene expression in young C. arabica plants

Young (4-month-old) seedlings of the cultivars Rubi MG1192, Icatú, Obatã and IAPAR59 (I59) of *C. arabica *[96] were planted (0.7 m within plants and 3 m between rows) at the Cerrados Agricultural Research Center (Planaltina-Distrito Federal, Brazil 15°35'44"S - 47°43'52"W) of the Embrapa, in full sun conditions in December 2007 and cultivated with (I) or without (NI) irrigation [76]. For the irrigated (I) condition, water was supplied by sprinklers (1.5 m height) organised in the field to perform uniform irrigation. Soil water content was controlled using PR2 profile probes (Delta-T Devices Ltd), and regular irrigations were performed to always maintain the water content above 0.27 cm^3 ^H_2_O cm^-1^. The points of analysis corresponded to the rainy (U, unstressed) and dry (WS, water-stress) seasons (Table 3).

#### The effects of water stress on RBCS1 gene expression in adult C. arabica plants

Adult (8 year old) *C. arabica *cv. Rubi and I59 plants were grown at the Cerrados Center in full sun conditions under continuous irrigation (I) or irrigation suspension (NI) during the dry season in 2008. The points of analysis were before (U1, unstressed), during (WS, water-stress) and after (U2, unstressed) the irrigation suspension period (Table 3). Irrigation conditions were identical to those described for young plants.

### Sample analysis and preparation

For both *C. arabica and C. canephora*, water stress levels were evaluated by measuring predawn leaf water potentials (*Ψ*_pd_) with a Scholander-type pressure chamber (Table 5) using fully expanded leaves (8-15 cm long) from the third or fourth pair from the apex of plagiotropic branches localised in the third upper part of the plant canopy. Leaves were collected between 3:00 and 5:00 am (night-time). For *C. arabica*, quantitative PCR (qPCR) experiments used leaves harvested at night (at the time of *Ψ*_pd _measurements) or between 10:00 and noon (daytime). For *C. canephora*, leaves were collected between 10:00 and noon for qPCR, Northern blot and 2-DE experiments. In that case, they were immediately frozen in liquid nitrogen and further conserved at -80°C before extraction.

### RNA isolation

Samples stored at -80°C were ground into a powder in liquid nitrogen, and total RNAs were extracted using the "Plant RNA Purification Reagent" (PRPR) method (Invitrogen). Around 50 mg of powder was added to 500 μl of PRPR buffer, mixed vigorously for 2 min at 25°C and then centrifuged (16000 × *g*, 2 min, 4°C). After the addition of 5 M NaCl (100 μl) and chloroform (300 μl) to the supernatant, the sample was centrifuge as previously described. One volume of isopropanol was further added to the supernatant. After incubation at 25°C for 30 min, nucleic acids were precipitated by centrifugation (16000 × *g*, 30 min, 4°C), and the pellet was dried and dissolved in 40 μl of RNAse-free water and stored at -20°C. RNA quantification was performed using a NanoDrop™ 1000 Spectrophotometer (Thermo Scientific, Waltham, MA, USA).

### Northern blot experiments

Fifteen micrograms of total RNA were fractionated on a 1.2% (w/v) agarose gel containing 2.2 M formaldehyde in MOPS buffer. Equal amounts of RNA samples were loaded and controlled by the abundance of the 26S and 18S rRNA on gels stained with ethidium bromide. The *CcRBCS1 *[GenBank:GT649534] and *CcUBQ10 *[GenBank:GT650583] probes were amplified by conventional PCR using universal primers from the plasmid harboring the corresponding EST sequences, and labelled by random priming with α-^32^P-dCTP (GE Healthcare) as previously described [97]. RNAs were transferred to Hybond N+ membranes followed by hybridisation at 65°C in modified Church and Gilbert buffer (7% SDS, 10 mM EDTA, 0.5 M sodium phosphate pH 7.2) and washed at 65°C in 2 × standard saline citrate (SSC; 1 × = 150 mM sodium chloride and 15 mM sodium citrate, pH 7.0), 0.1% SDS (2 × 15 min), with a final stringent wash in 0.1 × SSC, 0.1% SDS (2 × 15 min). Membranes were exposed with BAS-MS 2340 IP support, and the data was acquired using a Fluorescent Image Analyzer FLA-3000 (Fujifilm Life Science). When necessary, membranes were stripped and tested with a new probe.

### Cloning of the *CcRBCS1 *cDNA and gene sequences

The primer pair 18244 (*RBCS1-DNA*, Table 1), common to all cDNA of *RBCS1 *isoforms, was used to amplify *RBCS1 *cDNA sequences from the Rubi cultivar of *C. arabica *(pure *C. arabica *without introgression of *C. canephora*) and clone 14 of *C. canephora *var. Conilon, respectively. PCR was performed using a PTC-100 Thermocycler (MJ Research) with *Taq *Platinum DNA polymerase according to the supplier (Invitrogen) under the following conditions: initial denaturation at 94°C for 2 min followed by 40 cycles of 94°C for 30 s, Ta = 55°C for 30 s, and 72°C for 3 min, and a final extension step of 72°C for 7 min. The quality of the amplicons was verified by electrophoresis. PCR fragments were cleaned using the Wizard^® ^SV Gel and PCR Clean-Up System (Promega) and double-strand sequenced without cloning using the primers used for the PCR and the BigDye Terminator Sequencing Kit v3.1 chemistry on an ABI 3130*xl *Genetic Analyzer (Applied Biosystems). For the cloning of the *CcRBCS1 *gene, fresh leaves from clone 14 of *C. canephora *were collected in the greenhouse, immediately frozen in liquid nitrogen and used to extract genomic DNA as described previously [97]. The *CcRBCS1 *gene was amplified from genomic DNA (10 ng) using the primer pair 18244 (Table 1) and PCR conditions identical to those described before for the isolation of *CcRBCS1 *cDNA. The fragment obtained was cloned in pTOPO2.1 (Invitrogen) and double-strand sequenced.

### Multiple alignments

Multiple alignments of nucleic and protein sequences using sequences available from the online Sol Genomics Network (SGN, http://solgenomics.net/content/coffee.pl[56]) were obtained by the CLUSTALW program [98] followed by manual adjustment.

### Real time RT-PCR assays

To eliminate contaminant genomic DNA, samples were treated with RQ1 RNase-free DNase according to the manufacturer's instructions (Promega, Madison, WI, USA), and RNA quality was verified by agarose gel electrophoresis and visual inspection of the ribosomal RNA bands upon ethidium bromide staining. Synthesis of first strand cDNA was accomplished by treating 1 μg of total RNA with the ImProm-II™ Reverse Transcription System and oligo (dT_15_) according to the manufacturer's recommendations (Promega). The absence of contaminating genomic DNA in the cDNA preparations was checked by common PCR reaction using SUS10/SUS11 primer pair that spans introns 5 to 9 of the *CcSUS1 *gene (AJ880768), which encodes isoform 1 of the sucrose synthase from *C. canephora *[99]. RT-PCR was carried out using 1 μl of synthesised cDNA under conventional PCR conditions using a PTC-100 Thermocycler (MJ Research) with *GoTaq *DNA polymerase according to the supplier (Promega) with the following conditions: initial denaturation at 94°C for 2 min followed by 40 cycles of 94°C, Ta = 30 sec, 55°C for 30 sec and 72°C for 3 min and a final extension step of 72°C for 6 min. In such conditions, the amplification of a 667-bp fragment characterised the *CcSUS1 *cDNA, and the absence of corresponding genomic sequence is indicated by the lack of an amplicon at 1130-bp (data not shown).

Q-PCR was carried out with synthesised single-stranded cDNA as described above and using the protocol recommended for the use with 7500 Fast Real-Time PCR Systems (Applied Biosystems, Foster City, CA, USA). Preparations of cDNA were diluted (1:25 to 1:100) and tested by qPCR using *RBCS1*-specific primer pairs (Table 1) designed using Primer Express software (Applied Biosystems), which were preliminarily tested for their specificity and efficiency against a cDNA mix (data not shown). The qPCR was performed with 1 μl of diluted ss-cDNA and 0.2 μM (final concentration) of each primer in a final volume of 10 μl with 1 × SYBR green fluorochrome (SYBRGreen qPCR Mix-UDG/ROX, Invitrogen). The reaction mixture was incubated for 2 min at 50°C (Uracil DNA-Glycosylase treatment), then 5 min at 95°C (inactivation of UDGase), followed by 40 amplification cycles of 3 sec at 95°C and 30 sec at 60°C (annealing and elongation). Data were analysed using SDS 2.1 software (Applied Biosystems) to determine cycle threshold (Ct) values corresponding to the mean of triplicate samples. The specificity of the PCR products generated for each set of primers was verified by analysing the Tm (dissociation) of the amplified products. For each primer pair, PCR efficiencies (E) were estimated using absolute fluorescence data captured during the exponential phase of amplification of each reaction with the equation (1+E) = 10(-1/^slope^) [100]. Efficiencies were taken into account for all subsequent calculations. Expression values were expressed in relative quantification by applying the formula (1+E)^-ΔCt ^where ΔCt = Ct_mean _target gene - Ct_mean _reference gene. Gene expression levels were normalised (SDS 2.1 software) with the expression of the reference gene ubiquitin (*CcUBQ10*) for the experiments with *C. canephora *or glyceraldehyde-3-phosphate dehydrogenase (*GAPDH*) for other experiments [101].

### Protein extraction and analysis by two-dimensional gel electrophoresis (2-DE)

Total protein was extracted from leaves of clones 14 (drought-tolerant) and 22 (drought-susceptible) of *C. canephora *var. Conilon using a modified phenol/SDS method and further separated by two-dimensional gel electrophoresis (2-DE). The first dimension (isoelectric focalisation) was carried through using Immobilized pH gradient (IPG, pH 3-10 or pH 4-7) strips of 13 cm previously incubated (12 h, 20°C) with 500-1000 μg of protein and analysed using an Ettan IPGphor 3 Isoelectric Focusing system (GE Healthcare). The second dimension was made using a 11% SDS-polyacrylamide gel (PAGE) in a Hoefer SE 600 Ruby system (GE Healthcare) cooled at 12°C with 15 mA/gel for 45 min followed by 30 mA/gel for 180 min. Then, gels were stained using the colloidal (G-250) Coomassie blue method, and images were analysed with ImageMaster Software 2D Platinum 6.0. The normalised protein abundance of the RBSC1 isoforms was obtained from the relative spot volume expressed by percentage volume (%V), which was calculated from the gel images as the volume of a specific spot divided by the sum of the volume of all other spots present in the gel multiplied by 100. For protein sequence analysis, spots of interest were manually removed from gels, submitted to trypsin enzymatic treatment and analysed by mass spectrometry using a Maldi-TOF/TOF spectrometer (Bruker Daltonics). ImageMaster Platinum 6.0.

### Protein sequencing and identification

The proteins were identified by PMF ("Peptide Mass Fingerprinting") using PiumsGUI2.2 and MS/MS Ion Search using software X!Tandem. Obtained sequences were screened against the SOL Genomics Network and other coffee sequences available in public databases. The packages Trans-Proteomic Pipeline (TPP) and Scaffold were used to analyse protein data. MS and MS/MS analysis by MALDI TOF/TOF was performed for all protein spots that corresponded to RBSC1 isoforms. The results and sequences of the all identified peptides were further confirmed by *de novo *sequencing using FlexAnalysis software (Bruker Daltonics).

## Accession numbers

Sequence data from this article can be found in the Sol Genomics Network (SGN, http://solgenomics.net/content/coffee.pl[56]). The *CaRBCS1 *cDNA and corresponding gene sequences are available in the GenBank database under their respective accession numbers AJ419826 and AJ419827[58]. The *CcRBCS1 *(*CaCc*) cDNA and gene sequences reported here were deposited in the GenBank database under the accession numbers FR728242 and FR772689, respectively.

## Abbreviations

2-DE: two dimensional gel electrophoresis; EST: expressed sequence tag; MS: mass spectrometry; qPCR: quantitative polymerase chain reaction; RBCS: Rubisco small subunit; Rubisco: ribulose 1,5-bisphosphate carboxylase/oxygenase; SNP: single nucleotide polymorphism; Ta: temperature of annealing; ROS: reactive oxygen species; UTR: untranslated region; WS: water stress.

## Authors' contributions

LPF, GSCA, NGV, performed RNA extractions and gene expression studies by qPCR experiments with *C. arabica*, SE and FV qPCR expression studies of *C. canephora*. HJOR realized the experiment of 2-DE electrophoresis and MS sequencing, GCR and VAS measured predawn leaf water potentials and realized plant samplings. LPF and GSCA sequenced the *CcRBCS1 *and helped to analyze the data. TL, DP, GCR, LGEV, CM, ACA and PM conceived the study and elaborated the experimental design, data analysis and execution. PM and ACA drafted the manuscript. All authors read and approved the final version of manuscript.

## References

[B1] Pay E The market for organic and fair-trade coffee FAO Rome 2009

[B2] Davis AP Govaerts R Bridson DM Stoffelen P An annotated taxonomic conspectus of the genus *Coffea *(Rubiaceae) Bot J Linn Soc 2006 152 4 465 512 10.1111/j.1095-8339.2006.00584.x

[B3] Lashermes P Combes M-C Robert J Trouslot P D'Hont A Anthony F Charrier A Molecular characterization and origin of the *Coffea arabica *L genome Mol Gen Genet 1999 261 2 259 266 10.1007/s004380050965 10102360

[B4] Vidal RO Mondego JMC Pot D Ambrósio AB Andrade AC Pereira LFP Colombo CA Vieira LGE Carazzolle MF Pereira GAG A high-throughput data mining of single nucleotide polymorphisms in *Coffea *species expressed sequence tags suggests differential homeologous gene expression in the allotetraploid *Coffea arabica* Plant Physiol 2010 154 3 1053 1066 10.1104/pp.110.162438 20864545 PMC2971587

[B5] Lashermes P Andrzejewski S Bertrand B Combes MC Dussert S Graziosi G Trouslot P Anthony F Molecular analysis of introgressive breeding in coffee (*Coffea arabica *L.) Theor Appl Genet 2000 100 1 139 146 10.1007/s001220050019

[B6] DaMatta FM Exploring drought tolerance in coffee: a physiological approach with some insights for plant breeding Braz J Plant Physiol 2004 16 1 1 6

[B7] DaMatta FM Ramalho JC Impact of drought and temperature stress on coffee physiology and production: a review Braz J Plant Physiol 2006 18 1 55 81 10.1590/S1677-04202006000100006

[B8] Carr MKV The water relations and irrigation requirements of coffee Exp Agri 2001 37 1 1 36 10.1017/S0014479701001090

[B9] Silva EA Mazzafera P Brunini O Sakai E Arruda FB Mattoso LHC Carvalho CRL Pires RCM The influence of water management and environmental conditions on the chemical composition and beverage quality of coffee beans Braz J Plant Physiol 2005 17 2 229 238 10.1590/S1677-04202005000200006

[B10] Mazzafera P Garcia Salva TJ, Guerreiro Filho O, Thomaziello RA, Fazuoli LC Estresse hídrico e conseqüências na composição das sementes de café e qualidade da bebida Cafés de qualidade: aspectos tecnológicos, científicos e comerciais 2007 Instituto Agronômico Campinas 73 9021590758

[B11] Camargo AP Santinato R Cortez JG Aptidão climática para qualidade da bebida nas principais regiões cafeeiras de Arábica no Brasil18th Congresso Brasileiro de Pesquisas Cafeeiras 1992 Araxá, Minas Gerais, Brazil 70 74

[B12] Assad ED Pinto HS Zullo J Jr Ávila AMH Impacto das mudanças climáticas no zoneamento agroclimático do café no Brasil Pesq Agropec Bras 2004 39 11 1057 1064 10.1590/S0100-204X2004001100001

[B13] Chaves MM Effects of water deficits on carbon assimilation J Exp Bot 1991 42 1 1 16 10.1093/jxb/42.1.1

[B14] Cornic G Baker NR, Bowyer JR Drought stress and high light effects on leaf photosynthesis Photoinhibition of photosynthesis 1994 Bios Scientific Publishers, Oxford 297 331

[B15] Lawlor DW Marcelle R, Clijsters H, van Poucke M, Nijhoff M, Junk W Integration of biochemical processes in the physiology of water stressed plants Effects of stress on photosynthesis 1983 The Hague, The Netherlands 35 44

[B16] Lawlor DW Smirnoff N The effects of water deficit on photosynthesis Environment and Plant metabolism 1995 Bios Science Publishers. Oxford 129 160

[B17] Asada K The water-water cycle in chloroplasts: scavenging of active oxygens and dissipation of excess photons Annu Rev Plant Physiol Plant Mol Biol 1999 50 601 639 10.1146/annurev.arplant.50.1.601 15012221

[B18] DaMatta FM Rena AB Zambolin L Tolerância do café à seca Tecnologias de produção de café com qualidade 2001 Universidade Federal de Viçosa, Viçosa 65 100

[B19] DaMatta FM Hemantaranjan A Drought as a multidimensional stress affecting photosynthesis in tropical tree crops Advances in Plant Physiology 2003 5 Scientific Publishers, Jodhpur 227 265

[B20] Jensen RG Bahr JT Ribulose 1,5-bisphosphate carboxylase-oxygenase Ann Rev Plant Physiol 1977 28 379 400 10.1146/annurev.pp.28.060177.002115

[B21] Makino A Mae T Ohira K Relation between nitrogen and ribulose-1,5-bisphosphate carboxylase in rice leaves from emergence through senescence Plant Cell Physiol 1984 25 3 429 437

[B22] Feller U Anders I Mae T Rubiscolytics: fate of Rubisco after its enzymatic function in a cell is terminated J Exp Bot 2008 59 7 1615 1624 17975207 10.1093/jxb/erm242

[B23] Gutteridge S Gatenby AA Rubisco synthesis, assembly, mechanism, and regulation Plant Cell 1995 7 7 809 819 12242387 10.1105/tpc.7.7.809 PMC160870

[B24] Sheen J Metabolic repression of transcription in higher plants Plant Cell 1990 2 10 1027 1038 2136626 10.1105/tpc.2.10.1027 PMC159951

[B25] Krapp A Hofmann B Schafer C Stitt M Regulation of the expression of *rbcS *and other photosynthetic genes by carbohydrates: a mechanism for the 'sink regulation' of photosynthesis? Plant J 1993 3 6 817 828 10.1111/j.1365-313X.1993.00817.x

[B26] Parry MAJ Keys AJ Madgwick PJ Carmo-Silva AE Andralojc PJ Rubisco regulation: a role for inhibitors J Exp Bot 2008 59 7 1569 1580 18436543 10.1093/jxb/ern084

[B27] Dean C Pichersky E Dunsmuir P Structure, evolution, and regulation of *RbcS *genes in higher-plants Annu Rev Plant Physiol Plant Mol Biol 1989 40 415 439 10.1146/annurev.pp.40.060189.002215

[B28] Spreitzer RJ Role of the small subunit in ribulose-1,5-bisphosphate carboxylase/oxygenase Arch Biochem Biophys 2003 414 2 141 149 10.1016/S0003-9861(03)00171-1 12781765

[B29] Rizhsky L Liang H Mittler R The combined effect of drought stress and heat shock on gene expression in tobacco Plant Physiol 2002 130 3 1143 1151 10.1104/pp.006858 12427981 PMC166635

[B30] Bartholomew DM Bartley GE Scolnik PA Abscisic acid control of *rbcS *and *cab *transcription in tomato leaves Plant Physiol 1991 96 1 291 296 10.1104/pp.96.1.291 16668167 PMC1080748

[B31] Williams J Bulman MP Neill SJ Wilt-induced ABA biosynthesis, gene expression and down-regulation of *rbcS *mRNA levels in *Arabidopsis thaliana* Physiol Plant 1994 91 2 177 182 10.1111/j.1399-3054.1994.tb00416.x

[B32] Vu JCV Allen LH Jr Boote KJ Bowes G CO_2 _enrichment delays a rapid, drought-induced decrease in Rubisco small subunit transcript abundance J Plant Physiol 1999 155 1 139 142

[B33] Hayano-Kanashiro C Calderón-Vázquez C Ibarra-Laclette E Herrera-Estrella L Simpson J Analysis of gene expression and physiological responses in three mexican maize landraces under drought stress and recovery irrigation PLoS ONE 2009 4 10 e7531 10.1371/journal.pone.0007531 19888455 PMC2766256

[B34] Rizhsky L Liang H Shuman J Shulaev V Davletova S Mittler R When defense pathways collide. The response of *Arabidopsis *to a combination of drought and heat stress Plant Physiol 2004 134 4 1683 1696 10.1104/pp.103.033431 15047901 PMC419842

[B35] Lawlor DW Cornic G Photosynthetic carbon assimilation and associated metabolism in relation to water deficits in higher plants Plant Cell Environ 2002 25 2 275 294 10.1046/j.0016-8025.2001.00814.x 11841670

[B36] Reddy AR Chaitanya KV Vivekanandan M Drought induced responses of photosynthesis and antioxidant metabolism in higher plants J Plant Physiol 2004 161 11 1189 1202 10.1016/j.jplph.2004.01.013 15602811

[B37] Majumdar S Ghosh S Glick BR Dumbroff EB Activities of chlorophyllase, phosphoenolpiruvate carboxylase and ribulose 1,5-bisphosphate carboxylase in the primary leaves of soybean during senescence and drought Physiol Plant 1991 81 4 473 480 10.1111/j.1399-3054.1991.tb05087.x

[B38] Parry MAJ Andralojc PJ Madgwick PJ Khan S Lea PJ Medrano H Keys AJ Rubisco activity: effects of water stress Ann Bot 2002 89 7 833 839 10.1093/aob/mcf103 12102509 PMC4233807

[B39] Ali GM Komatsu S Proteomic analysis of rice leaf sheath during drought stress J Proteome Res 2006 5 2 396 403 10.1021/pr050291g 16457606

[B40] Lawlor DW Limitation to photosynthesis in water stressed leaves: stomata *vs *metabolism and role of ATP Ann Bot 2002 89 7 871 885 10.1093/aob/mcf110 12102513 PMC4233810

[B41] Demirevska K Simova L Vassileva V Feller U Rubisco and some chaperone protein responses to water stress and rewatering at early seedling growth of drought sensitive and tolerant wheat varieties Plant Growth Regul 2008 56 2 97 106 10.1007/s10725-008-9288-1

[B42] Medrano H Parry MAJ Socias X Lawlor DW Long term water deficit inactivates Rubisco in subtertanean clover Ann Appl Biol 1997 131 3 491 501 10.1111/j.1744-7348.1997.tb05176.x

[B43] Demirevska K Simova-Stoilova L Vassileva V Vaseva I Grigorova B Feller U Drought-induced leaf protein alterations in sensitive and tolerant wheat varieties Gen App Plant Physiol 2008 34 1-2 79 102

[B44] Houtz L Portis AR Jr The life of ribulose 1,5-bisphosphate carboxylase/oxygenase-posttranslational facts and mysteries Arch Biochem Biophys 2003 414 2 150 158 12781766 10.1016/s0003-9861(03)00122-x

[B45] Ferrão RG Fonseca AFA Silveira JSM Ferrão MAG Bragança SM EMCAPA 8141 - Robustão Capixaba, variedade clonal de café conilon tolerante à seca, desenvolvida para o estado do Espírito Santo Rev Ceres 2000 273 555 560

[B46] DaMatta FM Chaves ARM Pinheiro HA Ducatti C Loureiro ME Drought tolerance of two field-grown clones of *Coffea canephora* Plant Sci 2003 164 1 111 117 10.1016/S0168-9452(02)00342-4

[B47] Lima ALS DaMatta FM Pinheiro HA Totola MR Loureiro ME Photochemical responses and oxidative stress in two clones of *Coffea canephora *under water deficit conditions Environ Exp Bot 2002 47 3 239 247 10.1016/S0098-8472(01)00130-7

[B48] Meinzer FC Saliendra NZ Crisosto CH Carbon isotope discrimination and gas exchange in *Coffea arabica *during adjustment in different soil moisture regimes Aust J Plant Physiol 1992 19 2 171 184 10.1071/PP9920171

[B49] Silva EA DaMatta FM Ducatti C Regazzi AJ Barros RS Seasonal changes in vegetative growth and photosynthesis of Arabica coffee trees Field Crops Res 2004 89 2-3 349 357 10.1016/j.fcr.2004.02.010

[B50] Kanechi M Uchida NU Yasuda T Yamaguchi T Photosynthetic responses of *Coffea arabica *L to chronic dehydration during leaf development and after leaf maturation in unshaded and shaded conditions Jap J Trop Agric 1991 35 84 91

[B51] Kanechi M Uchida NU Yasuda T Yamaguchi T Non-stomatal inhibition associated with inactivation of Rubisco in dehydrated coffee leaves under unshaded and shaded conditions Plant Cell Physiol 1996 37 4 455 460

[B52] De Kochko A Akaffou S Andrade AC Campa C Crouzillat D Guyot R Hamon P Ming R Mueller LA Poncet V Tranchant-Dubreuil C Hamon S Kader JC, Delseny M Advances in *Coffea *genomics Advances in botanical research 2010 53 Oxford, Academic Press 23 53

[B53] Vieira LGE Andrade AC Colombo CA Moraes AAH Metha A Oliveira AC Labate CA Marino CL Monteiro-Vitorello CB Monte DC Giglioti E Kimura ET Romano E Kuramae EE Lemos EGM Almeida ERP Jorge EC Barros EVSA da Silva FR Vinecky F Sawazaki HE Dorry HFA Carrer H Abreu IN Batista JAN Teixeira JB Kitajima JP Xavier KG Lima LM Camargo LEA Pereira LFP Coutinho LL Lemos MVF Romano MR Machado MA Costa MMC Grossi de Sá MF Goldman MHS Ferro MIT Tinoco MLP Oliveira MC Sluys MAV Shimizu MS Maluf MP Eira MTS Guerreiro Filho O Arruda P Mazzafera P Mariani PDSC Oliveira RL Harakava R Balbao SF Tsai SM Mauro SMZ Santos SN Siqueira WJ Costa GGL Formighieri EF Carazzolle MF Pereira GAG Brazilian coffee genome project: an EST-based genomic resource Braz J Plant Physiol 2006 18 1 95 108 10.1590/S1677-04202006000100008

[B54] Lin CW Mueller LA Mc Carthy J Crouzillat D Petiard V Tanksley SD Coffee and tomato share common gene repertoires as revealed by deep sequencing of seed and cherry transcripts Theor Appl Genet 2005 112 1 114 130 10.1007/s00122-005-0112-2 16273343 PMC1544375

[B55] Poncet V Rondeau M Tranchand C Cayrel A Hamon S de Kochko A Hamon P SSR mining in coffee tree EST databases: potential use of EST-SSRs AS marker across *Coffea *genus Mol Gen Genet 2006 276 5 436 449 10.1007/s00438-006-0153-5 16924545

[B56] Mueller LA Solow TH Taylor N Skwarecki B Buels R Binns J Lin C Wright MH Ahrens R Wang Y Herbst EV Keyder ER Menda N Zamir D Tanksley SD The SOL Genomics Network A comparative resource for Solanaceae biology and beyond Plant Physiol 2005 138 3 1310 1317 10.1104/pp.105.060707 16010005 PMC1176404

[B57] Lashermes P Andrade AC Etienne H Moore H, Ming R Genomics of coffee, one of the world's largest traded commodities Genomics of Tropical Crop Plants 2008 Springer 203 226

[B58] Marraccini P Courjault C Caillet V Lausanne F Lepage B Rogers WJ Deshayes A Rubisco small subunit of *Coffea arabica*: cDNA sequence, gene cloning and promoter analysis in transgenic tobacco plants Plant Physiol Biochem 2003 41 1 17 25 10.1016/S0981-9428(02)00004-9

[B59] Keegstra K Olsen LJ Theg SM Chloroplastic precursors and their transport across the envelope membranes Annu Rev Plant Physiol Plant Mol Biol 1989 40 471 501 10.1146/annurev.pp.40.060189.002351

[B60] Pinheiro HA DaMatta FM Chaves ARM Fontes EPB Loureiro ME Drought tolerance in relation to protection against oxidative stress in clones of *Coffea canephora *subjected to long-term drought Plant Sci 2004 167 6 1307 1314 10.1016/j.plantsci.2004.06.027

[B61] Marraccini P Da Silva VA Elbelt S Guimarães BLS Loureiro ME DaMatta FM Ferrão MAG Da Fonseca AFA Da Silva F Andrade AC Analise da expressão de genes candidatos para a tolerância a seca em folhas de clones de *Coffea canephora *var Conillon, caracterizados fisiologicamente Proceedings of the V Simpósio de Pesquisa dos Cafés do Brasil 2007 Aguas de Lindoia (SP), Brasil

[B62] Kicheva MI Tsonev TD Popova LP Stomatal and non-stomatal limitations to photosynthesis in two wheat cultivars subjected to water stress Photosynthetica 1994 30 1 107 116

[B63] Dean C Pichersky E Dunsmuir P Structure, evolution, and regulation of *rbcS *genes in higher plants Ann Rev Plant Physiol Plant Mol Biol 1989 40 415 439 10.1146/annurev.pp.40.060189.002215

[B64] Leroy T De Bellis F Legnate H Kanamura E Gonzales G Pereira LF Andrade AC Charmetant P Montagnon C Cubry P Marraccini P Pot D de Kochko A Improving quality of African Robustas: QTL for agronomic and quality related traits in *Coffea canephora* Tree Genet Genomes10.1007/s11295-011-0374-6

[B65] Anthony F Bertrand B Quiros O Wilches A Lashermes P Berthaud J Charrier A Genetic diversity of wild coffee (*Coffea arabica *L.) using molecular markers Euphytica 2001 118 1 53 65 10.1023/A:1004013815166

[B66] Anthony F Combes C Astorga C Bertrand B Graziosi G Lashermes P The origin of cultivated *Coffea arabica *L varieties revealed by AFLP and SSR markers Theor Appl Genet 2002 104 5 894 900 10.1007/s00122-001-0798-8 12582651

[B67] Petitot AS Lecouls AC Fernandez D Sub-genomic origin and regulation patterns of a duplicated *WRKY *gene in the allotetraploid species *Coffea arabica* Tree Genet Genomes 2008 3 4 379 390

[B68] Cordell HJ Detecting gene-gene interactions that underlie human diseases Nat Rev Genet 2009 10 6 392 404 19434077 10.1038/nrg2579 PMC2872761

[B69] Fiévet JB Dillmann C de Vienne D Systemic properties of metabolic networks lead to an epistasis-based model for heterosis Theor Appl Genet 2010 120 2 463 473 10.1007/s00122-009-1203-2 19916003 PMC2793392

[B70] Lashermes P Combes MC Topart P Graziosi G Bertrand B Anthony F Sera T, Soccol CR, Pandey A, Roussos S Molecular breeding in coffee (*Coffea arabica*) Coffee, Biotechnology and quality 2000 Kluwer Academic Publishers 101 112

[B71] Bertrand B Guyot B Anthony F Lashermes P Impact of *Coffea canephora *gene introgression on beverage quality of *C arabica* Theor Appl Genet 2003 107 3 387 394 10.1007/s00122-003-1203-6 12750771

[B72] Privat I Bardil A Bombarely Gomez A Severac D Dantec C Fuentes I Mueller L Joët T Pot D Foucrier S Dussert S Leroy T Journot L de Kochko A Campa C Combes MC Lashermes P Bertrand B The 'PUCE CAFE' project: the first 15K coffee microarray, a new tool for discovering candidate genes correlated to agronomic and quality traits BMC Genomics 2011 12 5 10.1186/1471-2164-12-5 21208403 PMC3025959

[B73] Bardil A Combes MC Lashermes P Bertrand B Gene expression divergences between the allopolyploid *Coffea arabica *and its diploids relatives appear environment-dependant Abstracts of Plant and Animal Genomes XVIIIth Conference 2010 San Diego, CA (USA), W157

[B74] Chen ZJ Genetic and epigenetic mechanisms for gene expression and phenotypic variation in plant polyploids Annu Rev Plant Biol 2007 58 377 340 10.1146/annurev.arplant.58.032806.103835 17280525 PMC1949485

[B75] Bauer D Biehler K Fock H Carrayol E Hirel B Migg A Becker T A role for cytosolic glutamine synthetase in the remobilisation of leaf nitrogen during water stress in tomato Physiol Plant 1997 99 2 241 248 10.1111/j.1399-3054.1997.tb05408.x

[B76] Rodrigues GC Rojas JSD Roupsard O Leroy T Pot D Moreira MZ Verdeil JL Dauzat J Jourdan C Andrade AC Marraccini P Preliminary results on phenotypic plasticity of coffee (*Coffea arabica *cv Rubi and Iapar59) plants in response to water constraint under field conditions Proceedings of the 23rd International Scientific Colloquium on Coffee 2010 Bali, International Scientific Association on Coffee, Paris, CDROM-PB731

[B77] Gilmartin PM Sarokin L Memelink J Chua N-H Molecular light switches for plant genes Plant Cell 1990 2 5 369 378 2152164 10.1105/tpc.2.5.369 PMC159894

[B78] Trethewey RN ap Rees T The role of the hexose transporter in the chloroplasts of *Arabidopsis thaliana *L Planta 1994 195 2 168 174

[B79] Geiger DR Shieh WJ Yu XM Photosynthetic carbon metabolism and translocation in wild-type and starch-deficient mutant *Nicotiana sylvestris *L Plant Physiol 1995 107 2 507 514 12228378 10.1104/pp.107.2.507 PMC157154

[B80] Pilgrim ML McClung CR Differential involvement of the circadian clock in the expression of genes required for ribulose-1,5-bisphosphate carboxylase/oxygenase synthesis, assembly, and activation in *Arabidopsis thaliana* Plant Physiol 1993 103 2 553 564 12231961 10.1104/pp.103.2.553 PMC159015

[B81] Cheng SH Moore BD Seemann JR Effects of short- and long-term elevated CO_2 _on the expression of ribulose-1,5-bisphosphate carboxylase/oxygenase genes and carbohydrate accumulation in leaves of *Arabidopsis thaliana *(L.) Heynh Plant Physiol 1998 116 2 715 723 10.1104/pp.116.2.715 9489018 PMC35131

[B82] Praxedes SC DaMatta FM Loureiro ME Ferrão MAG Cordeiro AT Effects of long-term soil drought on photosynthesis and carbohydrate metabolism in mature robusta coffee (*Coffea canephora Pierre varkouillou*) leaves Environ Exp Bot 2006 56 3 263 273 10.1016/j.envexpbot.2005.02.008

[B83] Lilley JM Ludlow MM McCouch SR O'Toole JC Locating QTL for osmotic adjustment and dehydration tolerance in rice J Exp Bot 1996 47 9 1427 1436 10.1093/jxb/47.9.1427

[B84] Fu BY Xiong JH Zhu LH Zhao XQ Xu HX Gao YM Li YS Xu JL Li ZK Identification of functional candidate genes for drought tolerance in rice Mol Genet Genomics 2007 278 6 599 609 10.1007/s00438-007-0276-3 17665216

[B85] Xiong JH Fu BY Xu HX Li YS Proteomic analysis of PEG-simulated drought stress responsive proteins of rice leaves using a pyramiding rice line at the seedling stage Botanical Studies 2010 51 2 137 145

[B86] Webber AN Nie GY Long SP Acclimation of photosynthetic proteins to rising atmospheric CO_2_ Photosynthesis Res 1994 39 3 413 425 10.1007/BF00014595 24311133

[B87] Marraccini P Ramos HJO Vieira LGE Ferrão MAG da Silva FR Taquita JA Bloch C Jr Andrade AC Study of drought tolerance mechanisms in coffee plants by an integrated analysis Proceedings of the 22nd International Scientific Colloquium on Coffee 2008 Campinas, International Scientific Association on Coffee, Paris, CDROM-B212

[B88] Herppich WB Peckmann K Responses of gas exchange, photosynthesis, nocturnal acid accumulation and water relations of *Aptenia cordifolia *to short-term drought and rewatering J Plant Physiol 1997 150 4 467 474

[B89] Nogués S Baker NR Effects of drought on photosynthesis in Mediterranean plants grown under enhanced UV-B radiation J Exp Bot 2000 51 348 1309 1317 10.1093/jexbot/51.348.1309 10937707

[B90] DaMatta FM Loos RA Silva EA Loureiro ME Limitations to photosynthesis in *Coffea canephora *as a result of nitrogen and water availability J Plant Physiol 2002 159 9 975 981 10.1078/0176-1617-00807

[B91] Freire LP Vieira NG Vinecky F Alves GSC Leroy T Pot D Elbelt S Marques T Rodrigues GC Marraccini P Andrade AC Expression analysis and nucleic polymorphism of candidate genes for drought tolerance in coffee Proceedings of the 23rd International Scientific Colloquium on Coffee 2010 Bali, International Scientific Association on Coffee, Paris, CDROM-B202

[B92] Lashermes P Cros J Marmey P Charrier A Use of random amplified DNA markers to analyse genetic variability and relationships of Coffea species Genet Resour Crop Ev 1993 40 2 91 99 10.1007/BF00052639

[B93] Sera GH Sera T de Batista Fonseca IC Ito DS Resistance to leaf rust in coffee cultivars Coffee Science 2010 5 1 59 66

[B94] Fazuoli LC Carvalho CHS Carvalho GR Guerreiro-Filho O Pereira AA Bartholo GF Moura WM Silvarolla MB Braghini MT Carvalho CHS Cultivares de café arábica de porte alto Cultivares de Café: origem, características e recomendações 2008 Embrapa Café, Brasilia 227 254

[B95] Silva VA Antunes WC Guimarães BLS Paiva RMC Silva VF Ferrão MAG DaMatta FM Loureiro ME Physiological response of Conilon coffee clone sensitive to drought grafted onto tolerant rootstock Pesq Agropec Bras 2010 45 5 457 464 10.1590/S0100-204X2010000500004

[B96] Carvalho CHS Fazuoli LC Carvalho GR Guerreiro-Filho O Pereira AA de Almeida SR Matiello JB Bartholo GF Sera T Moura WM Mendes AMG de Rezende JC da Fonseca AFA Ferrão MAG Ferrão RG Nacif AP Silvarolla MB Braghini MT Carvalho CHS Cultivares de café arábica de porte baixo Cultivares de Café: origem, características e recomendações 2008 Embrapa Café, Brasilia 157 226

[B97] Geromel C Ferreira LP Cavalari AA Pereira LFP Guerreiro SMC Pot D Leroy T Vieira LGE Mazzafera P Marraccini P Biochemical and genomic analysis of sucrose metabolism during coffee (*Coffea arabica*) fruit development J Exp Bot 2006 57 12 3243 3258 10.1093/jxb/erl084 16926239

[B98] Thompson JD Higgins DG Gibson TJ CLUSTALW: improving the sensitivity of progressive multiple sequence alignment through sequence weighting, position specific gap penalties and weight matrix choice Nucleic Acids Res 1994 22 22 4673 4680 10.1093/nar/22.22.4673 7984417 PMC308517

[B99] Leroy T Marraccini P Dufour M Montagnon C Lashermes P Sabau X Ferreira LP Jourdan I Pot D Andrade AC Glaszmann JC Vieira LGE Piffanelli P Construction and characterization of a *Coffea canephora *BAC library to study the organization of sucrose biosynthesis genes Theor Applied Genet 2005 111 6 1032 1041 10.1007/s00122-005-0018-z 16133319

[B100] Ramakers C Ruijter JM Deprez RH Moorman AF Assumption-free analysis of quantitative real-time polymerase chain reaction (PCR) data Neurosci Lett 2003 339 1 62 66 10.1016/S0304-3940(02)01423-4 12618301

[B101] Barsalobres-Cavallari CF Severino FE Maluf MP Maia IG Identification of suitable internal control genes for expression studies in *Coffea arabica *under different experimental conditions BMC Mol Biol 2009 10 1 10.1186/1471-2199-10-1 19126214 PMC2629470

